# A Tandem Oligonucleotide Approach for SNP-Selective RNA Degradation Using Modified Antisense Oligonucleotides

**DOI:** 10.1371/journal.pone.0142139

**Published:** 2015-11-06

**Authors:** Dorota Magner, Ewa Biala, Jolanta Lisowiec-Wachnicka, Elzbieta Kierzek, Ryszard Kierzek

**Affiliations:** Institute of Bioorganic Chemistry Polish Academy of Sciences, 61-704 Poznan, Noskowskiego, 12/14, Poland; University of Edinburgh, UNITED KINGDOM

## Abstract

Antisense oligonucleotides have been studied for many years as a tool for gene silencing. One of the most difficult cases of selective RNA silencing involves the alleles of single nucleotide polymorphisms, in which the allele sequence is differentiated by a single nucleotide. A new approach to improve the performance of allele selectivity for antisense oligonucleotides is proposed. It is based on the simultaneous application of two oligonucleotides. One is complementary to the mutated form of the targeted RNA and is able to activate RNase H to cleave the RNA. The other oligonucleotide, which is complementary to the wild type allele of the targeted RNA, is able to inhibit RNase H cleavage. Five types of SNPs, C/G, G/C, G/A, A/G, and C/U, were analyzed within the sequence context of genes associated with neurodegenerative disorders such as Alzheimer’s disease, Parkinson’s disease, ALS (Amyotrophic Lateral Sclerosis), and Machado-Joseph disease. For most analyzed cases, the application of the tandem approach increased allele-selective RNA degradation 1.5–15 fold relative to the use of a single antisense oligonucleotide. The presented study proves that differentiation between single substitution is highly dependent on the nature of the SNP and surrounding nucleotides. These variables are crucial for determining the proper length of the inhibitor antisense oligonucleotide. In the tandem approach, the comparison of thermodynamic stability of the favorable duplexes WT RNA-inhibitor and Mut RNA-gapmer with the other possible duplexes allows for the evaluation of chances for the allele-selective degradation of RNA. A larger difference in thermodynamic stability between favorable duplexes and those that could possibly form, usually results in the better allele selectivity of RNA degradation.

## Introduction

Ribonucleic acids (RNA) are involved in different biological processes within the cell. The presence of altered, pathogenic variants of RNA often disrupts the normal course of cellular metabolism, what could be caused by an invalid RNA length or structure due to the mutations. Various approaches were tested to inhibit the expression of pathogenic RNA. Antisense oligonucleotides (ASOs) were the first promising tools for this purpose and, over time, ribozymes, decoys, aptamers and short interfering RNAs were developed [1–4].

In 1978, Zamecnik and Stephenson showed for the first time the inhibition of Rous sarcoma viral RNA translation by specific oligodeoxynucleotides [5]. To date, the precise and unambiguous origins of the inhibitory activities of ASOs are not well defined. This lack of detailed knowledge also concerns other approaches based on application of ribozymes, aptamers and interfering oligonucleotides (RNAi). ASOs are the smallest molecule with the potential to silence expression; thus, the origins of their therapeutic activities could be the easiest to solve.

Currently, there are two major models for the mechanism of ASO silencing [6]. One is based on the formation of a DNA/RNA duplex that promotes RNase H activation and cleavage of the RNA strand. It was observed that a 6–8 nucleotide long DNA oligomer is sufficient to activate RNase H [7, 8]. Another mechanism is based on the hybridization of the ASO to RNA regions important for biological functions, causing steric hindrance.

Studies of modified oligonucleotides have shown that a synthetic nucleic acid, the gapmer, can efficiently act as an antisense molecule by activating ribonuclease H, followed by mRNA cleavage [9]. A gapmer is formed by a 5–8 nucleotide DNA core (the gap) and both sides are flanked by modified nucleotides [7, 8]. The gapmer DNA fragment is responsible for activating RNase H, and the adjacent modified fragments increase its resistance to cellular nucleases and the thermodynamic stability of the gapmer/target RNA duplex. It was shown that flanking the DNA core with a LNA-2'OMeRNA-LNA fragment is optimal, as this modulation significantly enhances its chemical stability in human serum and improves the thermodynamic stability of duplex formation (E. Biala, R. Kierzek, unpublished data).

In a context of genomes, single nucleotide polymorphisms (SNPs) are often imperceptible changes mainly due to a well-functioning DNA repair mechanisms and the degeneracy of genetic code. SNPs are the most common type of genetic variation in the human genome, occurring on average every 100–300 nucleotides. SNPs can be distinguished by transitions and transversions and the transition-to-transversion ratio for *de novo* mutations in humans is approximately 1.7. Moreover, certain types of transitions and transversions occur more frequently than others. In the human genome, the most common are C/T (= G/A) substitutions among transitions and G/C (= C/G) among transversions [10–12].

Spontaneous or induced DNA and RNA sequence changes can cause diseases, alter the cellular response to drugs or pathogens, and, finally, they may be useful molecular diagnostic genetic markers. Most genetic diseases are polygenic; however, there are known some monogenic units caused by single substitutions that are inherited in a dominant or recessive manner, and may be autosomal or X-linked. The manner of inheritance usually determines the extent of their occurrence within the population. Neurodegenerative disorders such Alzheimer’s or Parkinson’s diseases have rare Mendelian versions that are inherited in a dominant manner, which themselves are not necessarily direct therapeutic targets due to their low incidence. However, they can provide important clues about the underlying disease pathways that function in the population [13–17]. In the case of heterozygotes, where wild type and mutant RNA are produced simultaneously, there is theoretically a possibility of silencing the mutant RNA and obtaining a healthy phenotype.

**Fig 1:**
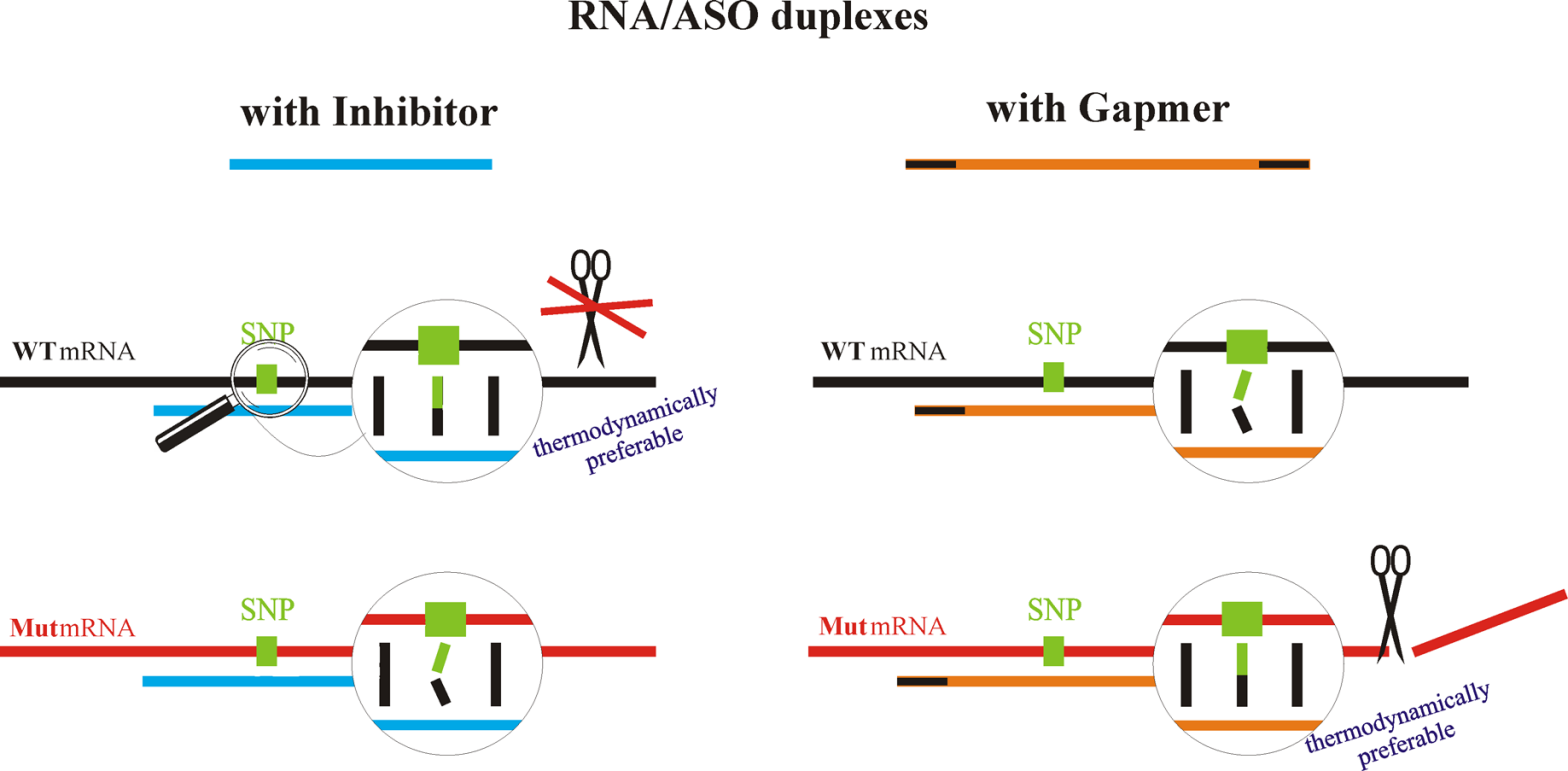
Schematic of the tandem antisense oligonucleotide approach. WT and Mut mRNA can interact with the gapmer or inhibitor, resulting in the formation of the most favorable thermodynamically duplexes (WT RNA/inhibitor and Mut RNA/gapmer). Possible to form duplexes of RNA variants with inhibitor antisense oligonucleotide (blue) and gapmer antisense oligonucleotide (orange-black) are presented. ASO molecules are designed in a way that gapmer preferentially binds the mutated RNA, promoting its cleavage with RNase H, while inhibitor preferentially hybridize to the wild type RNA, protecting it from RNase H activity.

Herein, a new approach to improve the performance of SNP selectivity for ASOs is proposed (Fig 1). Based on the thermodynamics of nucleic acid duplexes and the mechanism of ASO silencing, mutant-directed RNA hydrolysis by RNase H was achieved. The approach involves the simultaneous use of two oligonucleotides called tandem ASOs (S1 Table). One oligonucleotide (the active antisense or gapmer) is complementary to the mutated form of the target RNA, which has a single nucleotide mismatch with the wild type target RNA; this form is capable of activating RNase H to cleave the mutant RNA. The second oligonucleotide (the passive antisense or inhibitor) is complementary to the wild type RNA allele, which has a single nucleotide mismatch with the mutant type of RNA; this form is capable of inhibiting RNase H cleavage. In presented approach, it is crucial to select the tandem ASOs in such a way that the ASO gapmer, based on thermodynamics, will preferentially bind the mutant form of the target RNA (Mut RNA) and result in its cleavage. At the same time, the ASO inhibitor will preferentially bind the wild type allele of the target RNA (WT RNA) and, due to modifications in its composition (formed by 2’-O-methylated nucleotides), inhibits cleavage of the WT RNA by RNase H [18].

## Materials and Methods

### Selection of the target RNA molecules

The targets selected for these studies are fragments of human mRNA, in which point mutations can determine the development of neurodegenerative disorders, such as Alzheimer’s disease, Parkinson’s disease and ALS. The exception analyzed here is the case of Machado-Joseph disease, where the expansion of CAG repeats, and not the targeted G/C SNP of *SCA3* (spinocerebellar ataxia type 3) gene/mRNA, is pathogenic. Particular variants of the G/C SNP cosegregate with the wild type and mutated allele, respectively. Initial experiments were performed on RNA molecules from the fragment of the SCA 3 gene; however, the proposed conception generally refers to heterozygous mutations. Neurodegeneration is a complex process, which can be related to many genes. The particular SNPs were chosen because they are relatively most widespread among analyzed here neurodegenerative disease-causing point mutations as well as for the type of substitution. All of the mutations are inherited in autosomal dominant manner.

The first *in vitro* experiments concerned short, 13-nucleotide mRNA fragments, containing various types of point mutations placed in the center of the selected RNAs. The following SNPs were chosen: (*1*) C/G transversion in codon 692 of the *APP* (amyloid precursor protein) mRNA (rs63750671, A692G, here as C692G), associated with the development of Alzheimer’s disease; (*2*) G/C transversion occurring within codon 291 of the *SCA3* gene after the CAG trinucleotide repeats (rs12895357, G291R, here as G291C); (*3*) G/A transition in codon 717 of the *APP* mRNA (rs63750264, V717I, here as G717A), associated with the development of Alzheimer’s disease; (*4*) A/G transition in codon 693 of the *APP* mRNA (rs63750579, E693G, here as A693G), also associated with the development of Alzheimer’s disease; (*5*) G/A transition in codon 46 of the *SNCA* (α-synuclein) mRNA (rs104893875, E46K, here as G46A), associated with the development of Parkinson’s disease; (*6*) G/A transition in codon 53 of the *SNCA* (α-synuclein) mRNA (rs104893877, A53T, here as G53A), associated with the development of Parkinson’s disease; and (*7*) C/U transition in codon 4 of the *SOD1* (superoxide dismutase 1) mRNA (rs121912442, A4V, here as C4U), associated with the development of ALS.

### Oligonucleotides synthesis

All of the short RNAs (13-, 15- and 20-mers), ASO gapmers (13-, 15-, 17-, and 20-mers), ASO inhibitors (7- and 10-mers), and all PCR primers were synthesized on a BioAutomation MerMade12 DNA/RNA synthesizer using β-cyanoethyl phosphoramidite chemistry [19] and commercially available phosphoramidites (ChemGenes, GenePharma). The details of oligoribonucleotides deprotection and purification were described previously [20, 21].

### UV melting experiments

Oligonucleotides were melted in buffer containing 100 mM NaCl, 20 mM sodium cacodylate, and 0.5 mM Na_2_EDTA, pH 7. Only in the case of G/C SNP (G291C, *SCA3*), buffer containing 10 mM NaCl was used, because of inability to calculate thermodynamic parameters for 100 mM of sodium chloride. Oligonucleotide single strand concentrations were calculated from the absorbance above 80°C and single strand extinction coefficients were approximated by a nearest-neighbor model [22, 23]. Absorbance vs. temperature melting curves were measured at 260 nm with a heating rate of 1°C/min from 2 to 90°C on a Beckman DU 640 or JASCO V-650 spectrophotometer with a thermoprogrammer. The melting curves were analyzed and the thermodynamic parameters calculated from a two-state model with the program MeltWin 3.5 [24]. For most duplexes, the ΔH° derived from T_M_^-1^ vs. ln(C_T_/4) plots is within 15% of that derived from averaging the fits to individual melting curves, as expected if the two-state model is reasonable. The statistical analysis of thermodynamic data was carried out using MeltWin 3.5 software. The methods for determining sampling errors in ΔG°_37_, ΔH°, and ΔS° from the linear regression of T_M_^-1^ vs. ln(C_T_/4) plots using standard statistical analysis has been previously described [25]. Based on the results obtained for nine concentrations of a particular duplex, T_M_^-1^ vs. ln(C_T_/4), dependence was determined. The ΔG°_37_ value for the averaged 10^-4^ M concentration was calculated from this dependence and compared with that derived from averaging the fits to individual melting curves. Differences below 15% indicated that a two-state model is reasonable for the melting of the particular analyzed duplex.

### In vitro transcription

Long RNAs (99-mers) were obtained by *in vitro* transcription from the DNA template and were constructed in the PCR reaction. Two 74-nucleotide long, single-stranded DNA molecules, which were complementary to 18 nucleotides in length, were chemically synthesized and extended in PCR reactions to give 119 base-pairs of double-stranded DNA. The obtained molecule, containing a 99-nucleotide sequence of interest preceded by a T7 polymerase promoter sequence, was used as a template for *in vitro* transcription with the MEGAshortscript Kit from Ambion.

### RNase H assay

The pool of antisense gapmers complementary to 13-nucleotide of the WT and Mut RNAs was screened using the RNase H assay. RNAs were labeled at the 5’-end with γ^32^P ATP using T4 polynucleotide kinase (long RNA) or fluorescein (6-FAM) during the chemical synthesis of the oligonucleotides. Labeled RNA was mixed with ASO in an H-buffer in proportions that were optimized in separate experiments (1:10 for gapmers and 1:1 for inhibitors). The reaction mixture was denatured for 3 min at 80°C and then renatured at room temperature (RT) for 5 min to form duplexes. Next, 0.4 U of RNase H (*E*. *coli*) was added and the mixture was incubated for 20 min at 37°C. The time of RNase H digestion and the enzyme concentration were optimized in relation to the fully complementary RNA/gapmer duplexes. Cleavage products were analyzed on a 16% polyacrylamide gel with 7 M urea and then visualized by autoradiography. The digestion efficiency was evaluated from gel images using the MultiGauge 3.0 program (Fuji). The same assay was used to determine the kinetics of RNase H cleavage. A mixture of the appropriate duplex in H-buffer with ribonuclease H was incubated at 37°C and 10 μl aliquots were collected after 30 sec, 1, 5, 10 and 20 min of reaction. Twenty minutes was usually a sufficient time to complete RNA hydrolysis (S1 Fig). The hydrolysis of each sample was performed three times (n = 3). Values on the charts represent the mean of the hydrolysis efficiency, and error bars are standard deviation. The differences within replicates were statistically insignificant (P>0.05), whereas the significant differences between the hydrolysis of particular RNA variants (WT and Mut) are marked with an asterisk (P<0.05). Significance was determined using the online tool in-silico.net (t-Student test).

### Electrophoretic mobility shift assay (EMSA)

RNAs were labeled at the 5’-end with fluorescein (6-FAM) during chemical synthesis. Each RNA at constant concentration of 1 μM was mixed with ASO gapmer increasing concentration ranging from 10 nM to 1 μM in an H-buffer, containing 20 mM Tris-HCl, pH 7.8, 40 mM KCl, 8 mM MgCl_2_ and 1 mM DTT, to final volume of 10 μl. The samples were denatured at 95°C for 3 min and cooled at RT for 15 min. Next, hybridization was performed at 37°C for 30 min. Then, loading buffer containing 50% glycerol, 10 mM Tris-HCl, pH 7.8, Bromophenol Blue and xylene cyanol was added to final concentration of 10% glycerol, and samples were separated on 16% non-denaturing polyacrylamide gel in a TBM buffer containing 8 mM MgCl_2_. The electrophoresis was performed with cooling at RT and 20W for 6h. Samples after electrophoresis were visualized on gel using Fuji FLA-5100 fluorescence scanner, and analyzed in MultiGauge 3.0 software.

### HeLa cell line assays and RT-qPCR analysis

A day before transfection, *HeLa* cells were passaged to a 24-well plate and cultured in a standard RPMI 1640 medium containing 10% FBS (Invitrogen), 1x Antibiotic Animycotic solution (Sigma Aldrich), and 1x MEM vitamin solution (Sigma Aldrich). When the confluence reached around 90% per well, the cotransfection of *HeLa* cells with pEGFP expression plasmid containing cloned fragments of the genes of interest fused to the GFP protein, and gapmer or gapmer and inhibitor molecules, was carried out with Lipofectamine 2000 (Invitrogen), in a medium without antibiotics. After 24 hours of incubation, the cells were washed with phosphate-buffered saline (PBS) and viewed under a fluorescent microscope. Next, for the quantitative analysis of silencing, the total cellular RNA was extracted using the TRIzol^®^ method [26]. TRIzol was added in the amount of 500 μl per well and the plate was frozen until the next day for RNA isolation. Next, 0.5 μg of RNA was subjected to DNase I (Life Technologies) treatment. RNA quality was controlled by separation on 1.5% agarose gels. cDNA, which was the template for qPCR, was obtained in a reverse transcription reaction using the iScript cDNA Synthesis Kit (Bio-Rad). qPCR was performed on a CFX96 real-time PCR system (Bio-Rad) using iTaq SYBR Green Supermix (Bio-Rad) and 96-well clear plates. The level of GFP mRNA was quantified with the use of target gene primers GFPf 5’-GCTGACCCTGAAGTTCATC and GFPr 5’-GCTCCTGGACGTAGCCTTC, resulting in 164 bp product and normalized by human β-actin levels (reference gene primers: ACTf 5’-AGGCACCAGGGCGTGATG, ACTr 5’-TGATCTGGGTCATCTTCTCGC). The qPCR cycles are as follows: 95°C for 5 minutes; (95°C for 10 seconds and 60°C for 1 minute) for 40 cycles.

The statistical analysis of the qPCR results was performed with Bio-Rad CFX Manager 3.0 and OriginLab 8.0 software. Antisense oligonucleotides were tested in various concentration sets, and the transfection of each set was repeated two to five times (biological repeats). Quantitative PCR for each set was repeated three times (technical replicates). The results from the technical and biological repeats for particular samples were gathered for each concentration set transfected with WT or Mut constructs in order to determine the mean relative expression and its standard deviation (Bio-Rad CFX Manager 3.0). Differences between the obtained Cq values for particular repeated concentration sets higher than 0.5 were exluded from the analysis. Next, using ANOVA analysis (OriginLab 8.0), the normalized relative expression of GFP coupled with the WT and Mut RNA variants of analyzed genes was compared at the significance level of 0.05 for particular analyzed conditions. Statistically significant differences of the mean expression between WT and Mut alleles (P<0.05) were marked with an asterisk on the charts presenting qPCR results. Moreover, standard curves for target and reference genes were determined in order to control the PCR efficiency of each reaction, which ranged from 91 to 97% (S2 Fig).

## Results and Discussion

### Thermodynamic dependancies and their influence on RNase H cleavage of the RNA/ASO duplex

Non-canonical interactions of the nucleotides result in the diminishing of the thermodynamic stabilities of the duplexes due to the type of mismatches formed. Some single mismatches, such as G-U, G-A, G-G, or U-U, reduce duplex stabilities insignificantly, whereas A-A, C-C or U-C often cause significant changes to their thermodynamic stabilities [27–30]. The nature and orientation of the base pairs surrounding the mismatches can also influence destabilization [29, 31, 32]. Therefore, from the thermodynamic point of view, the type of substitution within the SNP site can substantially affect base pairing with the interacting oligonucleotides [28, 29]. As an example, compare the C/G transversion with the G/A transition. For the C/G transversion, the ASO that is complementary to the mutant allele G will form a C-C mismatch when hybridizing to the wild type C, which significantly reduces the duplex stability. In the case of the G/A transition, an antisense oligonucleotide that is complementary to the mutant A allele forms a pair of G-T with the wild type G allele, which is only slightly less stable than the canonical pair A-T [33, 34]. Generally, this means that the differentiation of SNP alleles in the G/A transition is much less likely than for the C/G transversion based on thermodynamics.

The main assumption of the presented approach was to differentiate the interactions of two types of oligonucleotides with WT and Mut RNA using an appropriate arrangement of 2'-O-methyl and LNA modified nucleotides within the tandem antisense oligonucleotides. As the distinction between canonical and non-canonical RNA-LNA base pairs is much stronger than between RNA-2’OMeRNA, RNA-RNA, or RNA-DNA, especially for internal positions [28, 31, 34, 35], the inhibitor was primarily expected to maintain the selectivity of the degradation of RNA SNP variants. In the mixture of tandem antisense oligonucleotides and both RNA SNP variants, the inhibitor should more favorably bind to the WT RNA and protect it from cleavage, whereas the gapmer should favorably bind to the Mut RNA, resulting in RNase H-induced cleavage. One of the most interesting thermodynamic parameters that characterize the tandem ASO approach is the difference in free energy (**ΔΔ**G°_37_) between the most stable duplexes formed in the mixture containing WT RNA, Mut RNA, gapmer, and inhibitor. These differences, obtained for all the analyzed RNA SNP types, confirmed the above assumption and were collected in Table 1. They were calculated on the basis of experimental data (S2 Table) obtained from UV meltings of particular duplexes.

**Table 1:** Comparison of the differences in stability of WT and Mut RNA duplexes with particular oligonucleotides, for all the analyzed SNP types. The greater the difference, the more selective hybridization was observed.

SNP	WT-Mut ΔΔG°_37_ (kcal/mol)		
	7-nt inhibitor	10-nt inhibitor	13-nt gapmer
C/G	4.69	3.96	-8.11
A/G	1.72	1.44	-5.31
G/A (APP717)	1.6	4.65	-0.5
G/A (SNCA46)	4.07	7.53	-3.36
G/A (SNCA53)	4.11	5.54	-2.7
C/U	2.29	0.48	-2.62
G/C	7.94	9.56	-9.76

All the duplexes melted in a two-state manner, indicating that the presence of 2’-O-methylRNA, LNA, and DNA residues do not disturb canonical base pairing [31, 36]. Two lengths of inhibitor molecule were tested to provide the most optimal and selective hybridization. The strongest differentiation of stability between duplexes formed by WT and Mut RNA with particular oligonucleotides was observed for both analyzed transversions types: G/C and C/G (Table 1). These types of SNPs impose the presence of a significantly destabilizing C-C mismatch within Mut RNA/inhibitor and WT RNA/gapmer duplexes, respectively. In both cases, slightly different consequences occur in the presence of RNase H. The strong impairment of the Mut RNA/inhibitor duplex causes preferential Mut RNA-gapmer hybridization, diminishing the pool of active gapmer available for the WT RNA and resulting in preferred WT RNA-inhibitor hybridization. On the other hand, even strong destabilization of the single nucleotide interactions within the WT RNA/gapmer duplex does not necessarily disturb the RNase H cleavage of WT RNA.

**Fig 2:**
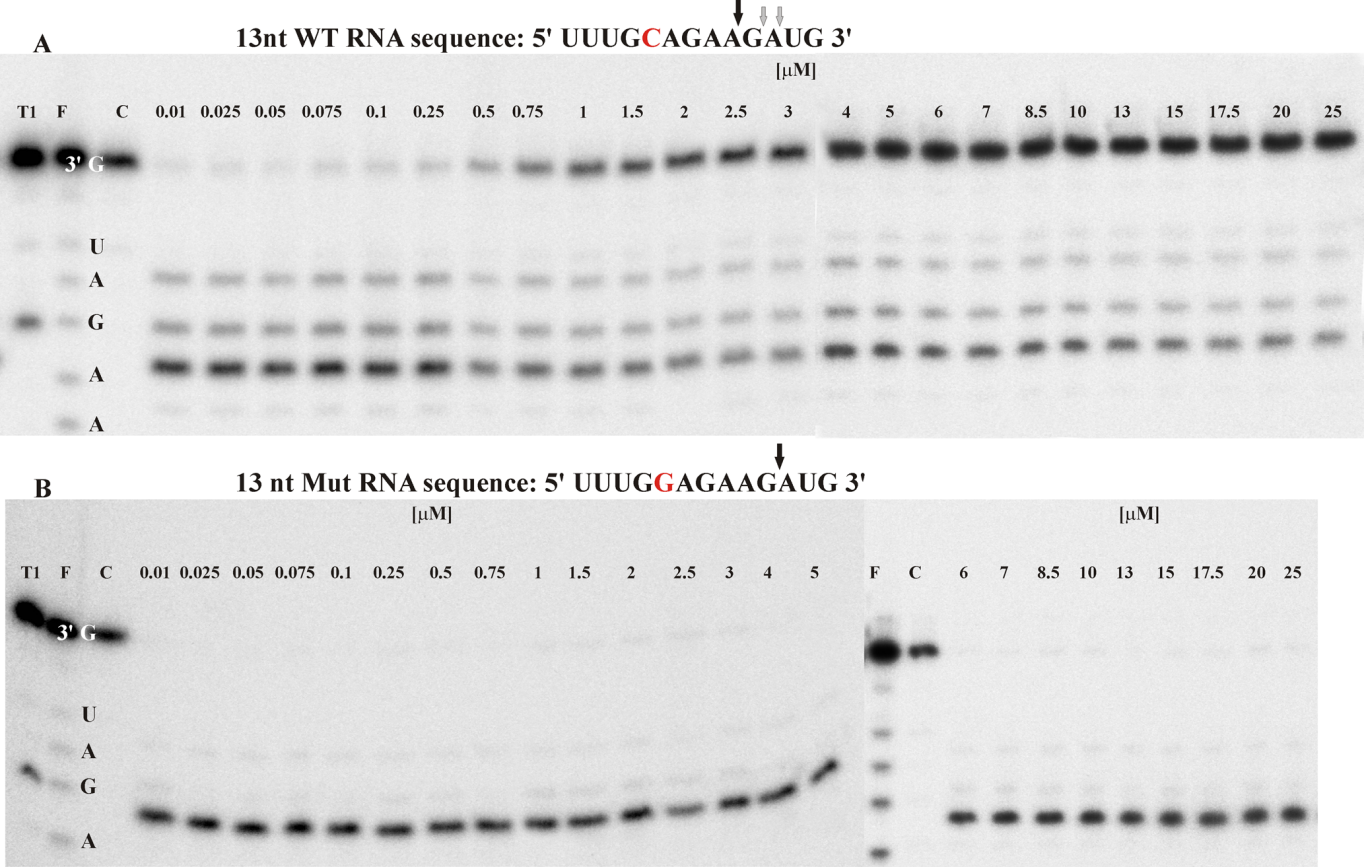
The RNase H assay results for G291C SNP within *SCA3* mRNA. **(A)** kinetics of the WT and Mut RNAs cleavage in the presence of the gapmer (fKM) and the shorter (If1) or longer (If2) inhibitor, where black squares indicate degradation of WT RNA in the presence of the If1/fKM/Mut RNA mixture, red dots—degradation of WT RNA in the presence of the If2/fKM/ Mut RNA mixture, blue triangles—degradation of Mut RNA in the presence of the If1/fKM/WT RNA mixture and green triangles—degradation of Mut RNA in the presence of the If2/fKM/WT RNA mixture, **(B)** stability of the WT RNA (green bar) and Mut RNA (red bar) in the presence of: gapmer fKM only (first pair of bars from the left), gapmer fKM and short inhibitor If1 (second) or gapmer fKM and longer inhibitor If2 (third), and in the WT/Mut RNA/fKM/If1 mixture (fourth) and WT/Mut RNA/fKM/If2 mixture (fifth); statistically significant differences between the mean hydrolysis efficiency of the RNA variants (P<0.05) are marked with asterisk **(C,D)** Results of *HeLa* cells cotransfection with WT/Mut G291C-pEGFP constructs and different amounts of inhibitor and gapmer antisense oligonucleotides. qPCR results of tandem approach with **(C)** shorter inhibitor If1, **(D)** Longer inhibitor If2. Statistically significant differences between the mean of the RNA variants expression (P<0.05) are marked with asterisk.

**Fig 3:**
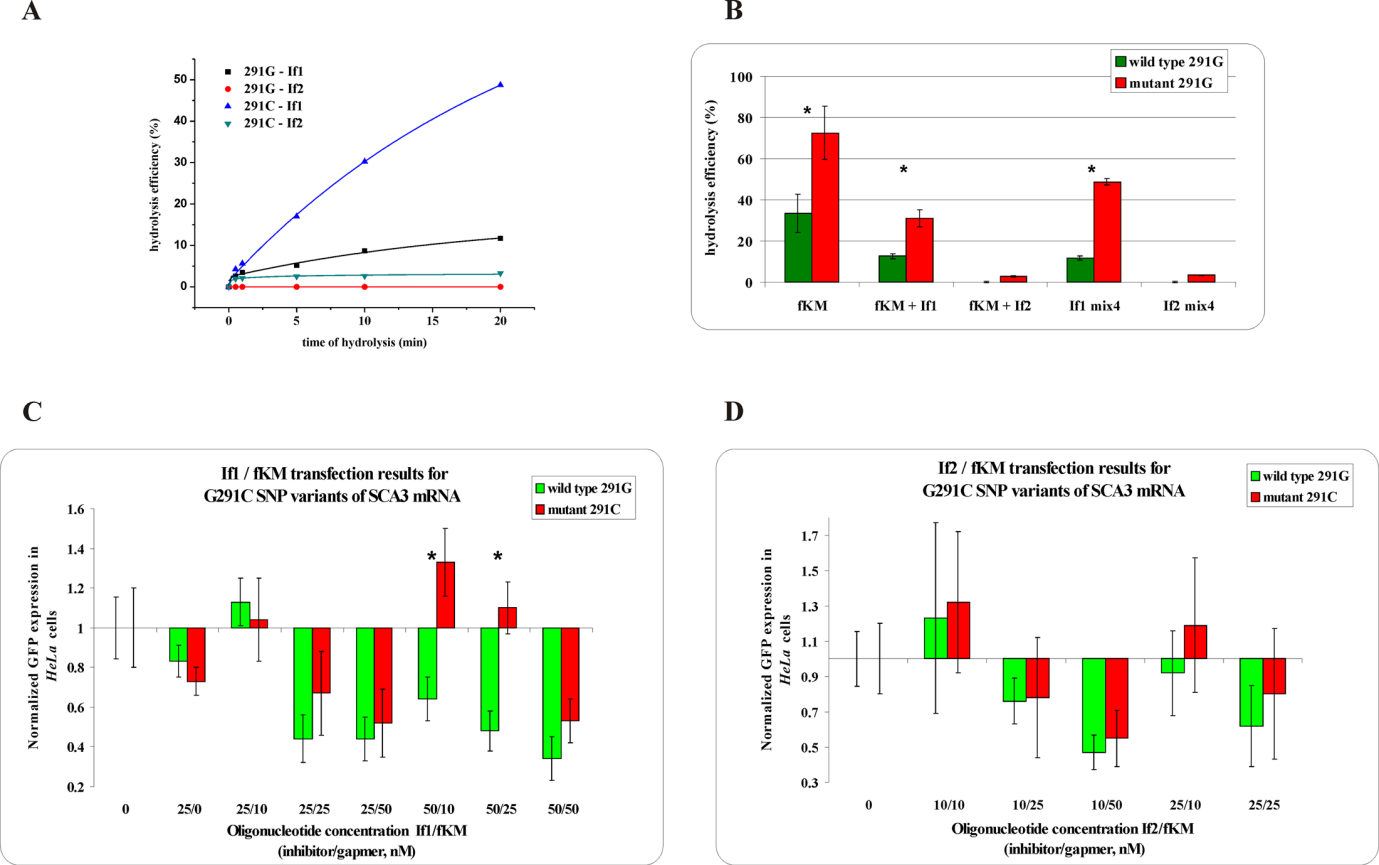
The RNase H assay results for C692G SNP within *APP* mRNA. **(A)** kinetics of the WT and Mut RNAs cleavage in the presence of the gapmer (bKM) and the shorter (Ib1) or longer (Ib2) inhibitor, where black squares indicate degradation of WT RNA in the presence of the Ib1/bKM/Mut RNA mixture, red dots—degradation of WT RNA in the presence of the Ib2/bKM/ Mut RNA mixture, blue triangles—degradation of Mut RNA in the presence of the Ib1/bKM/WT RNA mixture and green triangles—degradation of Mut RNA in the presence of the Ib2/bKM/WT RNA mixture, **(B)** stability of the WT RNA (green bar) and Mut RNA (red bar) in the presence of: gapmer bKM only (first pair of bars from the left), gapmer bKM and short inhibitor Ib1 (second) or gapmer bKM and longer inhibitor Ib2 (third), and in the WT/Mut RNA/bKM/Ib1 mixture (fourth) and WT/Mut RNA/bKM/Ib2 mixture (fifth); statistically significant differences between the mean hydrolysis efficiency of the RNA variants (P<0.05) are marked with asterisk, **(C,D)** Results of *HeLa* cells cotransfection with WT/Mut C692G-pEGFP constructs and different amounts of inhibitor and gapmer antisense oligonucleotides. qPCR results of tandem approach with **(C)** shorter inhibitor Ib1, **(D)** Longer inhibitor Ib2. Statistically significant differences between the mean of the RNA variants expression (P<0.05) are marked with asterisk.

Consequently, a sequence context of targeted SNP and the number of interacting nucleotides in a duplex play a key role in supporting or diminishing the effect of the mismatch. Therefore, it was impossible to clearly determine which of the inhibitors shorter or longer is more effective for selective hybridization to WT RNA and which could be applied universally for all of the SNP types. For the G/C SNP, analyzed in the context of *SCA3* mRNA, the better selectivity of Mut RNA degradation was achieved using the shorter inhibitor If1 (Fig 2). The elongation of the inhibitor by three nucleotides, two of which formed new G-C closing base pairs, significantly reduced the destabilizing effect of a mismatch and resulted in almost complete inhibition of digestion of both RNA forms (Fig 2). This effect was directly related to the sequence next to the SNP site, where 8 out of 10 base pairs are G-C pairs, and four of these are in its immediate vicinity. Moreover, the presence of the longer inhibitor resulted in the formation of very strong duplexes with WT RNA, which were stable in denaturing conditions (7 M urea, S3 Fig). In turn, for the opposite transversion C/G analyzed in the sequence context of *APP* mRNA, the longer inhibitor more efficiently protected WT RNA from RNase H digestion, without significant influence on Mut RNA cleavage (Fig 3B). In this case, the elongation of the inhibitor oligonucleotide by three nucleotides resulted in the formation of two new U-A closing base pairs which did not abolish the differentiating effect of the mismatch. Moreover, the duplex of Mut RNA 692G with an inhibitor, containing a G-G^L^ mismatch is two times less stable than the complementary duplex of WT RNA 692C with an inhibitor and four times less stable than the complementary duplex of Mut RNA with a gapmer, which determines the hybridization affinity of tandem oligonucleotides to particular RNAs. Also, Kd values obtained for duplexes of both RNA 692 variants with a gapmer indicate that there is three times more preference for the hybridization of a gapmer with Mut RNA than with the WT RNA variant (S3 Table).

**Fig 4:**
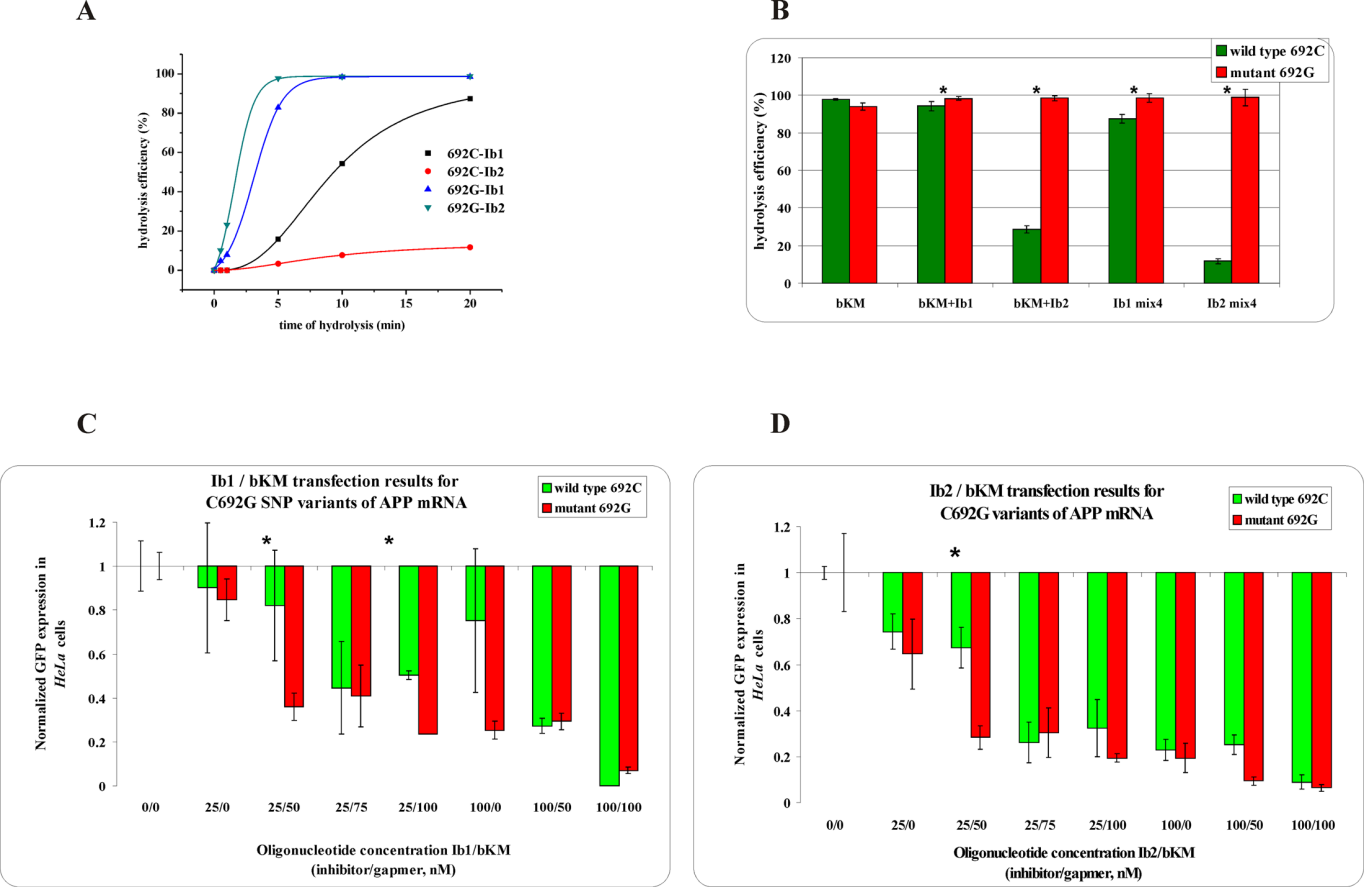
The RNase H assay results for G46A SNP within *SNCA* mRNA. **(A)** kinetics of the WT and Mut RNAs cleavage in the presence of the gapmer (kKM) and the shorter (Ik1) or longer (Ik2) inhibitor, where black squares indicate degradation of WT RNA in the presence of the Ik1/kKM/Mut RNA mixture, red dots—degradation of WT RNA in the presence of the Ik2/kKM/ Mut RNA mixture, blue triangles—degradation of Mut RNA in the presence of the Ik1/kKM/WT RNA mixture and green triangles—degradation of Mut RNA in the presence of the Ik2/kKM/WT RNA mixture, **(B)** stability of the WT RNA (green bar) and Mut RNA (red bar) in the presence of: gapmer kKM only (first pair of bars from the left), gapmer kKM and short inhibitor Ik1 (second) or gapmer kKM and longer inhibitor Ik2 (third), and in the WT/Mut RNA/kKM/Ik1 mixture (fourth) and WT/Mut RNA/kKM/Ik2 mixture (fifth). Statistically significant differences between the mean hydrolysis efficiency of the RNA variants (P<0.05) are marked with asterisk, **(C,D)** Results of *HeLa* cells cotransfection with WT/Mut G46A -pEGFP constructs and different amounts of inhibitor and gapmer antisense oligonucleotides. qPCR results of tandem approach with **(C)** shorter inhibitor Ik1, **(D)** Longer inhibitor Ik2. Statistically significant differences between the mean of the RNA variants expression (P<0.05) are marked with asterisk.

For the analyzed cases of G/A transitions, the thermodynamic differences between the stability of possible duplexes were significantly lower than in the case of the transversions presented above (Table 1). This SNP type thermodynamically facilitates the differentiation of the interactions of RNA variants with an inhibitor molecule (strongly stabilizing G-C^L^ pair versus strongly destabilizing A-C^L^ mismatch), but discrimination between duplexes formed by a gapmer (G-T mismatch versus A-T pair) is very poor. In all three analyzed cases, the Kd values obtained for RNA G/A variants of duplexes with a gapmer indicate no significant difference in the binding affinity to particular alleles. This fact confirmed that duplex discrimination between single G-T and A-T base pairs is hardly possible. Also, in these three cases of G/A transition, the SNP sequence context ultimately prejudges the final result. The highest differences in thermodynamic stability between duplexes of WT and Mut RNAs with both oligonucleotides were obtained for the G46A transition within *SNCA* mRNA, for which the application of the shorter inhibitor better discriminated between the degradation of RNA variants *in vitro* with RNase H and in *HeLa* cells (Fig 4 and S4 Fig).

The quantitative differences of the digestion efficiency of *in vitro* RNase H of WT and Mut RNAs are presented in S2 Table. Thermodynamically, the difference between the stabilities of the two preferred duplexes (WT RNA/inhibitor and Mut RNA/gapmer) substantially influences the duplex formation process in a competitive environment. Among all of the tested cases of SNPs, these differences were more pronounced when shorter inhibitors were applied, mainly because they involved fewer nucleotide residues in the interactions than the longer ones. Again, depending on the SNP type and its nucleotide context, these differences ranged from 13.3 to 1.7 kcal/mol when a shorter inhibitor was applied, and from 6.8 to 2.9 kcal/mol with the use of a longer inhibitor.

If the interactions of oligonucleotides with target RNAs within a living cell were only dependent on thermodynamics, the largest **ΔΔ**G°_37_ value would be related to the strongest SNP-selective RNase H cleavage. However, the results presented here indicate that the interactions within cells do not only follow the thermodynamic rules [37, 38]. In addition, stoichiometry turned out to be very important for high SNP selectivity *in vitro* and in *HeLa* cells.

### The stoichiometry optimization of RNase H and *HeLa* cells assays

**Fig 5:**
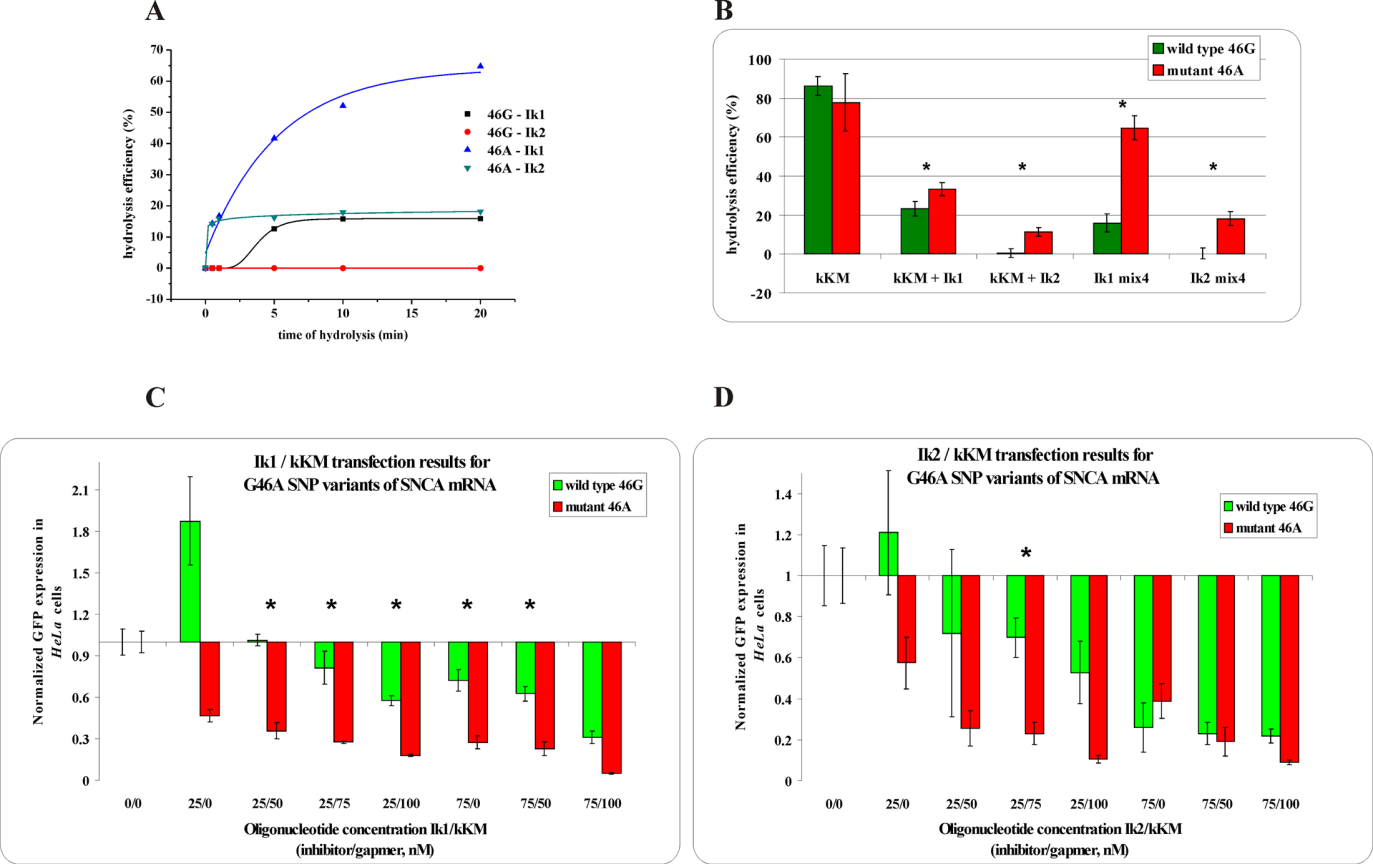
The influence of the shorter inhibitor Ib1 on the cleavage of RNA C692G transversion variants in the presence of bKM gapmer oligonucleotide. **(A)** wild type RNA allele C **(B)** mutated RNA allele G. The sequence and the cleavage sites of analyzed RNAs are presented above gel images. The concentrations of WT and/or Mut RNA variants and the gapmer were 100 nM and 10 nM, respectively. The concentration of the Ib1 inhibitor ranged from 0.01 to 25 μM, as indicated by numbers above the lines. RNase H assay of designed model RNAs usually resulted in 1–2 main products of cleavage and a few minor products. The cleavage pattern is characteristic for particular duplex and could differ depending on presence or absence of a mismatch. Abbreviations in the gel picture mean as follow: T1- RNase T1 cleavage products, F—formamide hydrolysis products, C—control sample. The control sample contained all the mixture components exept for gapmer oligonucleotide.

To select the optimal concentration of the inhibitor, a range of concentrations from 0.01–25 μM was tested. The results for C/G transversion are shown in Fig 5. Similar experiments were conducted for G/A and C/U transitions (S5 Fig). Based on quantitative gel analysis, the IC_50_ value was 599 nM for Ib1/WT 692C RNA (C/G transversion), but only 107 nM for Id1/WT 717G RNA (G/A transition). In the case of C/G transversion, it was shown that the presence of the shorter inhibitor Ib1 in the reaction mixture together with the Mut RNA has no influence on RNA degradation because the gapmer bKM forms a more thermodynamically stable (**Δ**G°_37_) duplex with the mutated RNA (-17.92 kcal/mol) than the competing Ib1/Mut RNA duplex (-4.60 kcal/mol). The opposite situation was observed for WT RNA, where, at 0.75 μM of Ib1, the inhibition of WT RNA cleavage increased significantly, reaching over 50%. With increasing concentrations of Ib1, a slow gradual inhibition of WT RNA degradation was observed. The maximum inhibition of WT RNA cleavage (78%) was reached at 20 μM of Ib1. The thermodynamic stabilities of Ib1/WT RNA (-9.29 kcal/mol) and bKM/WT RNA (-9.81 kcal/mol) duplexes are fairly similar, and thus the gapmer bKM can also bind WT RNA at low concentrations of Ib1. When the amount of the inhibitor Ib1 increased, most of the bKM/WT RNA duplex was converted into the Ib1/WT RNA duplex and the degradation of the WT RNA was limited. Based on studies performed with 1 μM of inhibitor, a final inhibitor concentration of ten-fold of the target RNA and one hundred-fold of the gapmer was selected. It was shown (S6 Fig) that at the chosen concentrations of inhibitors, Mut RNA was quantitatively digested, whereas WT RNA was completely stable in the presence of the longer inhibitor Ib2 and was 90% stable in the presence of the shorter inhibitor Ib1. The elongation of the WT RNA by two nucleotides allowed for the simultaneous and quantitative analysis of both target RNAs in one reaction using polyacrylamide gel electrophoresis (PAGE). The results are presented in Fig 3 and S7A Fig. The experiments show that the mixture of both RNAs, the gapmer bKMm and the longer inhibitor (Ib2) efficiently inhibits the gapmer and drives the cleavage of the WT RNA. After five minutes of hydrolysis, the mutated RNA was almost completely cleaved, whereas only approximately 5% of the WT RNA was degraded at that time. The application of the shorter inhibitor (Ib1) resulted in a less selective RNA cleavage, and after five minutes approximately 80% and 20% of the Mut and WT RNA were cleaved, respectively.

Comparisons of the stabilities of the oligonucleotide models of WT RNA and Mut RNA in mixtures containing two (RNA and gapmer), three (RNA, inhibitor, and gapmer), and four (both forms of RNA, inhibitor, and gapmer) oligonucleotides indicated the increasing potential for allele-selective RNA degradation (Fig 3B). The analysis shows that the presence of the gapmer by itself did not result in allele-specific cleavage of the target RNAs. However, in the presence of the longer inhibitor (Ib2), significant selectivity was observed and the Mut RNA was four times more accessible to cleavage. The shorter inhibitor (Ib1) basically had no influence on selectivity, and the observed cleavage rate was similar to reactions performed in the absence of an inhibitor. Allele selectivity seems to be even more efficient when the inhibitor Ib2 and both target RNAs are present in the reaction mixture simultaneously. For the shorter inhibitor (Ib1), selectivity was lower; however, as can be seen in Fig 3A, extending the time of RNase H treatment results only in diminishing the selectivity of WT and Mut RNA cleavage.

Preliminary *HeLa* transfection experiments were performed with each of the antisense oligonucleotides separately at concentrations of up to 100 nM. In the case of C/G transversion, the results obtained for transfection with inhibitors showed a significant influence on the expression of GFP for both alleles (S8A Fig for Ib1 and S8B Fig for Ib2, respectively). It was observed that at higher concentrations of Ib1 or Ib2 inhibitors, stronger but less SNP-selective GFP silencing was achieved. The transfection of *HeLa* cells with the gapmer bKM at 25, 50, 100, 200 and 300 nM also increased the non-allele-selective silencing of GFP (S8C Fig). For further experiments, the gapmer was used in concentrations ranging from 25–100 nM.

### Final results

The analysis targeting the stability of RNA variants in the presence of both inhibitors and gapmers was important for determining whether RNase H digestion results in a competitive environment are consistent with the thermodynamic preference for hybridization. Of course, the cellular environment is a much more complicated system in which competition for binding is much stronger due to molecular crowding and the multiplicity of possible non-specific interactions which hinder oligonucleotide-RNA hybridization.

#### *APP* gene

*HeLa* cells were transfected with a plasmid containing model WT and Mut RNAs at various concentrations of inhibitor Ib1 and gapmer bKM (Fig 3C) or inhibitor Ib2 and gapmer bKM (Fig 3D). Under these conditions, allele-selective GFP silencing was more efficient than without the inhibitor oligonucleotide. The silencing of both model RNAs are analyzed by GFP expression; therefore, transfection experiments were carried out separately for each plasmid. The results gathered in S2 Table showed that for specific concentrations of inhibitor and gapmer, the difference between the degradation of RNA variants was statistically significant. The expression of the Mut RNA was 40% lower than that of the WT RNA with tandem antisense oligonucleotides of 25 nM of Ib1 and 50 nM of bKM, and 27% lower using 25 nM of Ib1 and 100 nM of bKM (Fig 3C and S7B Fig). When the cotransfection was performed with Ib2 (at a concentration of 25 nM) and bKM (50 nM) in tandem, the level of expression of both RNA alleles differed by 39% (Fig 3D and S7C Fig, marked with an asterisk). A comparison of the results for this particular C/G transversion indicates that in *HeLa* cells, both the shorter and the longer inhibitor act with similar selectivity. However, the shorter one had a slightly wider range of effective concentrations when acting in tandem with the gapmer oligonucleotide.

**Fig 6:**
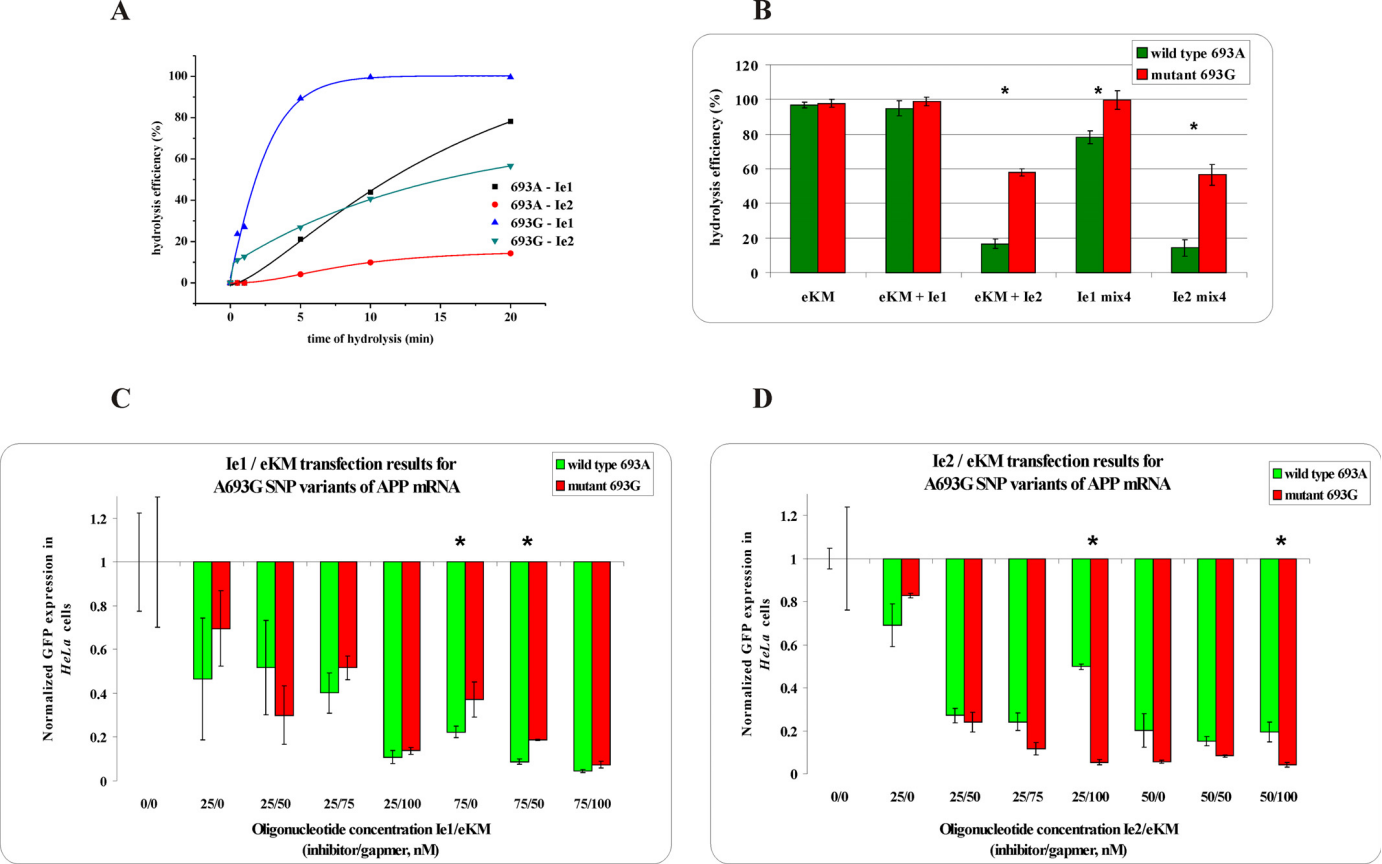
The RNase H assay results for A693G SNP within *APP* mRNA. **(A)** kinetics of the WT and Mut RNAs cleavage in the presence of the gapmer (eKM) and the shorter (Ie1) or longer (Ie2) inhibitor, where black squares indicate degradation of WT RNA in the presence of the Ie1/eKM/Mut RNA mixture, red dots—degradation of WT RNA in the presence of the Ie2/eKM/ Mut RNA mixture, blue triangles—degradation of Mut RNA in the presence of the Ie1/eKM/WT RNA mixture and green triangles—degradation of Mut RNA in the presence of the Ie2/eKM/WT RNA mixture, **(B)** stability of the WT RNA (green bar) and Mut RNA (red bar) in the presence of: gapmer eKM only (first pair of bars from the left), gapmer eKM and short inhibitor Ie1 (second) or gapmer eKM and longer inhibitor Ie2 (third), and in the WT/Mut RNA/eKM/Ie1 mixture (fourth) and WT/Mut RNA/eKM/Ie2 mixture (fifth). Statistically significant differences between the mean hydrolysis efficiency of the RNA variants (P<0.05) are marked with asterisk, **(C,D)** Results of *HeLa* cells cotransfection with WT/Mut A693G -pEGFP constructs and different amounts of inhibitor and gapmer antisense oligonucleotides. qPCR results of tandem approach with **(C)** shorter inhibitor Ie1, **(D)** Longer inhibitor Ie2. Statistically significant differences between the mean of the RNA variants expression (P<0.05) are marked with asterisk.

A substitution in the contiguous codon 693 of the *APP* gene A/G transition was more difficult for allele-selective degradation. *In vitro* experiments showed that the presence of the shorter inhibitor (Ie1) did not result in a decrease of WT RNA cleavage, whereas the presence of the longer inhibitor (Ie2) diminished cleavage by 80% and 40% for WT and Mut RNAs, respectively (Fig 6). Cotransfection of *HeLa* cells with the gapmer and shorter inhibitor (Ie1) significantly decreased the expression of Mut RNA and WT RNA, but the presence of the longer inhibitor (Ie2) resulted in an expression selectivity of 45% in favor of WT RNA. The results from RNase H and *HeLa* cell assays differed more between each other when the shorter inhibitor was applied, which could suggest an increased specifity of Ie2.

On the other hand, for the G/A transition in codon 717 of the APP mRNA, better SNP-selective RNA degradation was observed in the in vitro RNase H assay when the longer 10-nucleotide inhibitor Id2 was applied. In *HeLa* cells, however, no selective silencing was obtained for any of the inhibitors (S9E and S9F Fig). These results indicate that the key role for SNP-selective RNA degradation with tandem oligonucleotides appears to be their sequence, length, and concentration.

#### SNCA, SOD1, and SCA3 genes

Similar experiments in *HeLa* cells were performed for the G/C transversion in codon 291 of the *SCA3* mRNA (Fig 2), the G/A transition in codons 46 and 53 of the *SNCA* (α-synuclein) mRNA (Fig 4C and 4D and S9C and S9D Fig, respectively), and the C/U transition in codon 4 of the *SOD1* mRNA (S9A and S9B Fig). Detailed results were collected in S2 Table. For particular SNPs, various levels of selectivity were reported when using different inhibitor lengths, and also in the case of the same substitution type (3 examples of G/A transition).

For each of three analyzed G/A transition cases, slightly different dependencies were observed. The best allele selectivity for RNA degradation *in vitro*, as well as RNA silencing in *HeLa* cells, was obtained for the G46A substitution within the context of the *SNCA* mRNA. In comparison to the other two cases of G/A transition, both inhibitors and the kKM gapmer showed the strongest selectivity relative to their complementary RNA variants based on thermodynamic parameters. *In vitro*, the shorter inhibitor differentiated the efficiency of the hydrolysis of the two RNA variants by nearly 50%, providing better selectivity than the longer inhibitor, which strongly limited the efficiency of Mut RNA hydrolysis (Fig 4A and 4B, and S4A Fig). These results recurred in cellular conditions, under which a broader range of Ik1 concentrations were effective than for Ik2 (Fig 4C and 4D). The outcomes obtained for the G53A substitution showed that the shorter inhibitor also acted more effectively, promoting cleavage in favor of the Mut RNA *in vitro* with 83% selectivity (S9C Fig) and with 45% selectivity in *HeLa* cells for inhibitor/gapmer concentrations of 25 nM/100 nM (S10C Fig) The use of the longer inhibitor resulted in the significant reduction or even complete loss of allele selectivity in the analyzed concentration sets (S10D Fig). As the results for the three cases of G/A transitions were not consistent, the conclusion is that each case of SNP, including those of the same type, should be analyzed individually. The nucleotide context of SNP change is one of the key factors for effective inhibitor/gapmer cooperation resulting in SNP-selective RNA degradation using the tandem approach.

In the case of the C4U transition, the cleavage efficiency of WT RNA in the presence of the shorter inhibitor (Ia1) observed in *in vitro* experiments was reduced by 46% with no influence on Mut RNA degradation (S10A and S10B Fig). The longer inhibitor (Ia2) diminished WT RNA degradation by 75% and Mut RNA degradation by 12%. The results obtained from *HeLa* cells showed that in the presence of the shorter inhibitor (Ia1), no statistically significant selective silencing of the mutated RNA was observed, whereas the difference in the level of expression between WT and Mut RNA was only 27% for the longer inhibitor (Ia2).

*In vitro* RNase H assay results for G291C SNP within *SCA3* mRNA (Fig 2) showed that in the presence of only the gapmer, WT RNA is cleaved more efficiently than Mut RNA. The application of the shorter inhibitor If1 caused the strong inhibition of WT RNA degradation with no influence on the cleavage of Mut RNA. The longer inhibitor If2 totally disrupted the cleavage of both RNA alleles. However, *HeLa* cell experiments did not entirely confirm results observed *in vitro*. The wild type allele G was silenced slightly more efficiently than mutant C for both tested inhibitors, although the difference was not statistically significant in any of the tested concentrations (Fig 2C and 2D).

Generally, it seems that the effectiveness of the tandem antisense oligonucleotide approach is primarily dependent on the selective binding of the inhibitor to WT RNA, as a single mismatch within the duplex is mostly insufficient for RNase H activity differentiation. For SNP alleles where the inhibitor showed a similar binding affinity to WT and Mut RNA, allele-selective degradation was much more difficult to obtain. Additionally, for nucleotide substitutions resulting in relatively strong non-canonical base pairs, such as G-T, U-G, or G-G, the elongation of the inhibitor molecule for each subsequent nucleotide resulted in a significant reduction of its potential for selective binding.

### Significance of the tandem approach results

Allele-selective silencing of different genes is described for some neurodegenerative diseases inherited in a dominant manner, including Huntington disease, different types of spinocerebellar ataxias, Alzheimer’s disease, and Amyotrophic Lateral Sclerosis (ALS) [39–44]. For that purpose, siRNAs [43, 45–49] and ASOs [50–54] are commonly used. The direct cause of the disease (such as the expansion of trinucleotide repeats in *HTT* or *SCA3* genes) is often an inefficient target for allele differentiation; therefore, other sequence variations (such as short deletions or SNPs), which only remain in linkage disequilibrium with a particular allele, become a target for this strategy. Herein, almost all target SNPs are mutations directly associated with neurodegeneration resulting from protein structure changes related to the occurrence of SNP in its coding region. However, the differentiation of the allele’s expression level on the basis of a single mismatch occurring within siRNA duplexes or RNA/DNA duplexes of SNP variants of RNA is not as obvious and simple as it might seem.

Single mismatch was reported as a target for allele discrimination mostly with the use of RNA interference [39, 40, 42, 47, 55–57]. The data presented here showed that a single mismatch used in the tandem ASO oligonucleotide approach could be applied to the SNP-selective degradation of WT and Mut RNAs. The outcomes of the experiments demonstrated that the allele-selective degradation of RNAs differing by a single nucleotide was dependent on the nature of the nucleotide substitution (type of formed mismatch) within the target RNA. It is presumably correlated with differences in the thermodynamic stability of the fully complementary helix formed between the Mut RNA and the inhibitor or gapmer and the single mismatched duplex formed between the WT RNA and the inhibitor or gapmer. Thermodynamic measurements showed that the differences in the stabilities (**ΔΔ**G°_37_) of ASO duplexes ranged within 12.12 and -4.03 kcal/mol. The most optimal situation would be if the inhibitor preferentially bound to the WT RNA (inhibitor/WT RNA) while the gapmer bound to the Mut RNA (gapmer/Mut RNA) and promoted RNase H activity. Analysis of the obtained data also indicated that the largest difference in thermodynamic duplex stabilities occurs when the inhibitor is shorter. On average, the differences in thermodynamic stabilities were 7.3 and 2.4 kcal/mol for duplexes of the short inhibitor/WT RNA and gapmer/Mut RNA and the longer inhibitor/WT RNA and gapmer/Mut RNA duplexes, respectively.

The results presented above allow some conclusions to be drawn concerning the tandem oligonucleotide approach for SNP-selective RNA cleavage. The results summarized in S2 Table show that the presence of inhibitors enhanced selectivity by 1.5- to 15-fold for *in vitro* experiments and by 2- to 15-fold for *HeLa* cell experiments, with the exception of the RNA carrying the C4U *SOD1* SNP. Differentiation in the abilities of the SNP-selective cleavage of WT and Mut RNAs is somehow correlated with the thermodynamic stabilities of the duplexes formed by the inhibitors and gapmers with the WT and Mut RNAs. When the interaction of inhibitors and gapmers with the Mut RNA generated A-C and C-C mismatches, (C692G, G46A, G53A) the process of distinguishing WT and Mut RNA was more efficient. The other types of mismatches did not cause significant differences in RNA variant stability.

In the *HeLa* cell experiments, the observed selectivity of the tandem approach was less clear. Based on thermodynamic rules, the application of the shorter inhibitor should better differentiate its selective binding to WT and Mut RNAs. This dependency was observed mainly when the inhibitor hybridized to the Mut RNA, generating strongly destabilizing base pairs such as A-C (G/A transition), although it was not a rule for the C-C mismatch (C/G transversion) [28]. In the same context of inhibitor-formed duplexes, for relatively stable non-canonical base pairs such as G-T and U-G (transitions A693G and C4U), the longer inhibitor better differentiated allele hydrolysis efficiency *in vitro*. This result was likely associated with the stronger pairing of the inhibitor with the WT RNA and the lower affinity of the gapmer to the WT RNA in view of the intensely destabilizing A-C and C-A mismatches. The results obtained suggest that SNP sequence context and the type and place of nucleotide modification within the ASO are significant for allele-selective discrimination of pathogenic RNA as, in general, they control the final difference in total duplex stability. This difference is crucial during oligonucleotide competition for hybridization with particular RNA variants. Experiments *in vitro* containing model WT and Mut RNAs as well as both an inhibitor and a gapmer confirmed that the tandem approach significantly enhanced SNP-selective allele discrimination. *HeLa* cell experiments showed that the use of the shorter inhibitor resulted in allele-selective discrimination more often than the longer inhibitor. Certain differences in the selectivity of RNA inhibition which were observed between results obtained *in vitro* and in *HeLa* cells could be a consequence of the tertiary structure of the target RNA as well as its intermolecular interactions inside the living cell. These types of interactions usually play a minor role in *in vitro* conditions, which result in less molecular crowding and for which it is easier to determine and predict the behavior of molecules. The Dependency of ASO activity relative to target RNA structure has already been reported [58, 59] and is important for understanding differences that could potentially occur *in vitro* and in cell culture conditions. In cells, where ASO could interact not only with the designated fragment of target RNA but also with other RNAs present in its vicinity, adverse off-target effects could appear due to complementary and partially mismatched interactions, which was noted for ASOs and siRNAs [59–61]. The scale of non-specific effects depends on many factors including the accessibility of the target region within its structure. For all of the target RNAs which were analyzed in *HeLa* cells as molecules of 100 nucleotides, structure prediction was performed in RNAStructure 5.0, which did not indicate significant barriers for efficient ASO hybridization. For the same molecules analyzed *in vitro* in the RNase H assay, digestion results were comparable with those obtained for 13-nucleotide RNAs.

Based on the obtained results, some correlations between the thermodynamic stabilities of RNA duplexes and allele-selective degradation *in vitro* were noted. In most cases, the simultaneous presence of all cleavage reaction components (model RNAs, inhibitor, and gapmer) significantly enhanced SNP selectivity for RNA cleavage. Moreover, in certain cases, the application of the longer inhibitor results in better allele discrimination. For experiments in *HeLa* cells, the correlation between the thermodynamic parameters and allele-selective degradation is less obvious. It concerns not only the role of the longer and shorter inhibitors but also the reaction mixture itself, which contains many components that can interact with each other. The thermodynamic rules for nucleic acid folding were not determined for intracellular conditions and the crowding effect in cells. The contingency of other potential interactions can limit the thermodynamic dependence of the SNP-selective cleavage of target RNAs using tandem ASO oligonucleotides.

## Conclusions

The collective results presented here indicated that the tandem approach enhanced SNP selectivity between two RNA alleles *in vitro*; however, the observed selectivity was less efficient in *HeLa* cells. In the case of single substitutions, selectivity is highly dependent on the nature of the SNP and surrounding nucleotides. Significant differences in the thermodynamics of the duplexes formed by the inhibitor and gapmer with the WT and Mut RNAs often improved the SNP-selective degradation of Mut RNA. The tandem ASO approach is not a universal method, but taking into account the SNP type, length and sequence of 2’-O-methyl inhibitor oligonucleotide, as well as the nucleotide composition of the modified gapmer, the tandem ASO approach could be successfully applied to support the allele-selective degradation of some pathogenic RNA variants that differ by one nucleotide.

## Supporting Information

S1 Fig Kinetics of selected, complementary RNA/gapmer duplexes cleavage with RNase H. Most of the analyzed RNAs were totally degraded up to twenty minutes of RNase H hydrolysis. This could be seen also in the charts presenting RNase H hydrolysis efficiency depending on oligonucleotides content in the reaction mixture (Figs 3A, 4A, 5A and 6A and the upper charts in the S7 Fig. The exeption was additionally tested, as one of the controls, 717G-dKW duplex (WT RNA of APP G717A SNP with its complemetary gapmer antisense oligonucleotide dKW) which, probably because of the sequence, was digested five times slower.
(TIF)

S2 Fig Normalization of qPCR. Standard curves for GFP (target, upper chart—A) β-actin (reference, middle chart—B), and their superposition for relative gene expression normalization (lower chart—C). Parameters of the curves were determined with Bio-Rad CFX Manager 3.0 software. As the standard curves for target and reference genes run in parallel, expression changes were determined with direct method based of comparison of the normalized target expression with the control sample, which usually was transfection of plasmid constructs only.
(TIF)

S3 Fig Gel image after electrophoresis of G291C alleles cleavage of SCA3 RNA with RNase H and If1/fKM or If2/fKM antisense oligonucleotides. If2 inhibitor changes the WT RNA migration under denaturing gel conditions (stable duplex in 7M urea).
(TIF)

S4 Fig **(A)** Gel image after electrophoresis of G46A alleles cleavage of SNCA RNA with RNase H and Ik1/kKM or Ik2/kKM antisense oligonucleotides. **(B)** and **(C)** Fluorescence microscope images of *HeLa* cells (magnification 10x) after 24h cotransfection with WT/Mut G46A -pEGFP constructs and concentrations of inhibitor (shorter—B, and longer—C) and gapmer antisense oligonucleotides, that give statistically significant difference between both alleles expression.
(TIF)

S5 Fig The influence of the shorter inhibitor on the cleavage of G717A RNA variants (I) and C4U RNA variants (II) in the presence of the gapmer. (A) model WT RNA, (B) model Mut RNA. Numbers above the path refer to inhibitor concentration (μM), arrows indicate cleavage sites.
(TIF)

S6 Fig Stabilities of RNA C/G transversion variants in presence of gapmer and selected concentrations of the Ib1/Ib2 inhibitors. (A) WT 692C RNA and (B) Mutated 692G RNA.
(TIF)

S7 Fig (A) Gel image after electrophoresis of C692G alleles cleavage of APP RNA with RNase H and Ib1/bKM or Ib2/bKM antisense oligonucleotides. **(B)** and **(C)** Fluorescence microscope images of *HeLa* cells (magnification 10x) after 24h cotransfection with WT/Mut C692G -pEGFP constructs and concentrations of inhibitor (shorter—B, and longer—C) and gapmer antisense oligonucleotides, that give statistically significant difference between both alleles expression.
(TIF)

S8 Fig Level of GFP expression induced by only the (A) inhibitor Ib1, (B) inhibitor Ib2 and (C) gapmer bKM, after statistical analysis of qPCR results. Statistically significant difference (P<0.05) are marked by asterisks.
(TIF)

S9 Fig *In vitro* RNase H assay results for C4U (A), G717A (B) and G53A (C) transitions. Upper charts present kinetics of RNase H hydrolysis, lower charts—efficiency of hydrolysis depending on presence of reaction mixture components. Statistically significant differences between mean hydrolysis efficiency of WT and Mut RNA cleavage are marked with asterisk (P<0.05, based on t-Student test)
(TIF)

S10 Fig qPCR results for C4U (A-B), G53A (C-D), and G717A (E-F) transition variants differentiation. Statistically significant differences between mean normalized GFP expression of analyzed RNA variants are marked with asterisk (P<0.05)
(TIF)

S11 Fig Optimization of the antisense oligonucleotide concentration for *in vitro* RNase H assay. T1—RNase T1 cleavage of 692G RNA, F—formamide hydrolysis of 692G RNA, K—control sample without gapmer oligonucleotide (bKM concentration of 0)
(TIF)

S1 Table Antisense oligonucleotides sequences used for silencing (N^M^—2’-O-Me-RNA, N^L^—LNA, n—DNA). (PDF)

S2 Table Experimental data presenting thermodynamic parameters, efficiency of RNase H cleavage and transfection results for the best SNP selectivity for all tested SNPs. (PDF)

S3 Table Kd values for particular SNP variants of RNA/gapmer duplexes. (PDF)

S4 Table Sequences of analysed short RNA fragments. SNP site is bolded. (PDF)
