# Correction: A Tandem Oligonucleotide Approach for SNP-Selective RNA Degradation Using Modified Antisense Oligonucleotides

**DOI:** 10.1371/journal.pone.0163575

**Published:** 2016-09-19

**Authors:** Dorota Magner, Ewa Biala, Jolanta Lisowiec-Wachnicka, Elzbieta Kierzek, Ryszard Kierzek

The images for Figs 2–5 are incorrectly switched. The image that appears as Fig 2 should be Fig 5. The image that appears as Fig 3 should be Fig 2. The image that appears as Fig 4 should be Fig 3. The image that appears as Fig 5 should be Fig 4. The figure captions appear in the correct order.

**Fig 2:**
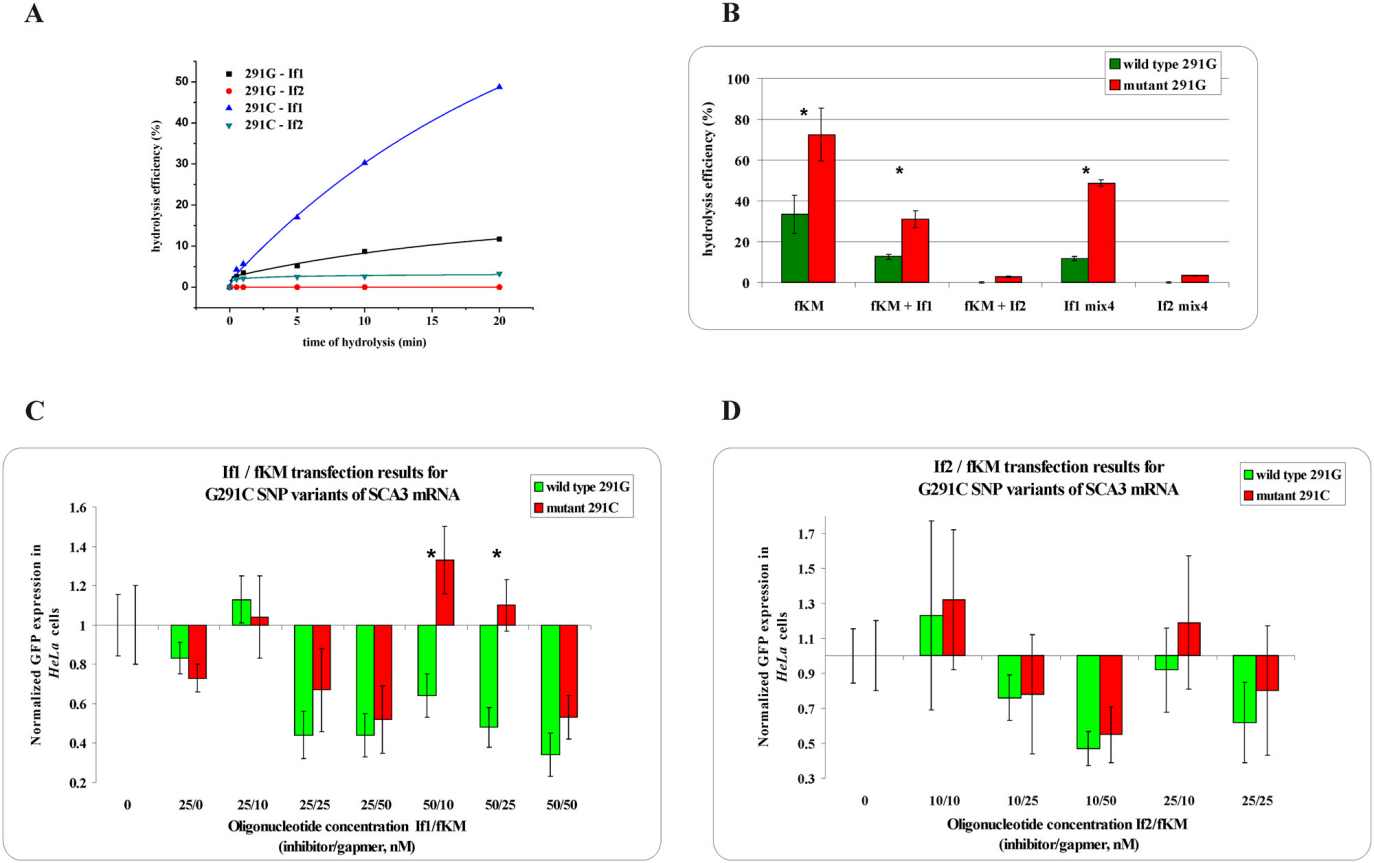
The RNase H assay results for G291C SNP within *SCA3* mRNA. ** (A)** kinetics of the WT and Mut RNAs cleavage in the presence of the gapmer (fKM) and the shorter (If1) or longer (If2) inhibitor, where black squares indicate degradation of WT RNA in the presence of the If1/fKM/Mut RNA mixture, red dots—degradation of WT RNA in the presence of the If2/fKM/ Mut RNA mixture, blue triangles—degradation of Mut RNA in the presence of the If1/fKM/WT RNA mixture and green triangles—degradation of Mut RNA in the presence of the If2/fKM/WT RNA mixture, **(B)** stability of the WT RNA (green bar) and Mut RNA (red bar) in the presence of: gapmer fKM only (first pair of bars from the left), gapmer fKM and short inhibitor If1 (second) or gapmer fKM and longer inhibitor If2 (third), and in the WT/Mut RNA/fKM/If1 mixture (fourth) and WT/Mut RNA/fKM/If2 mixture (fifth); statistically significant differences between the mean hydrolysis efficiency of the RNA variants (P<0.05) are marked with asterisk **(C,D)**Results of *HeLa* cells cotransfection with WT/Mut G291C-pEGFP constructs and different amounts of inhibitor and gapmer antisense oligonucleotides. qPCR results of tandem approach with **(C)** shorter inhibitor If1, **(D)** Longer inhibitor If2. Statistically significant differences between the mean of the RNA variants expression (P<0.05) are marked with asterisk.

**Fig 3:**
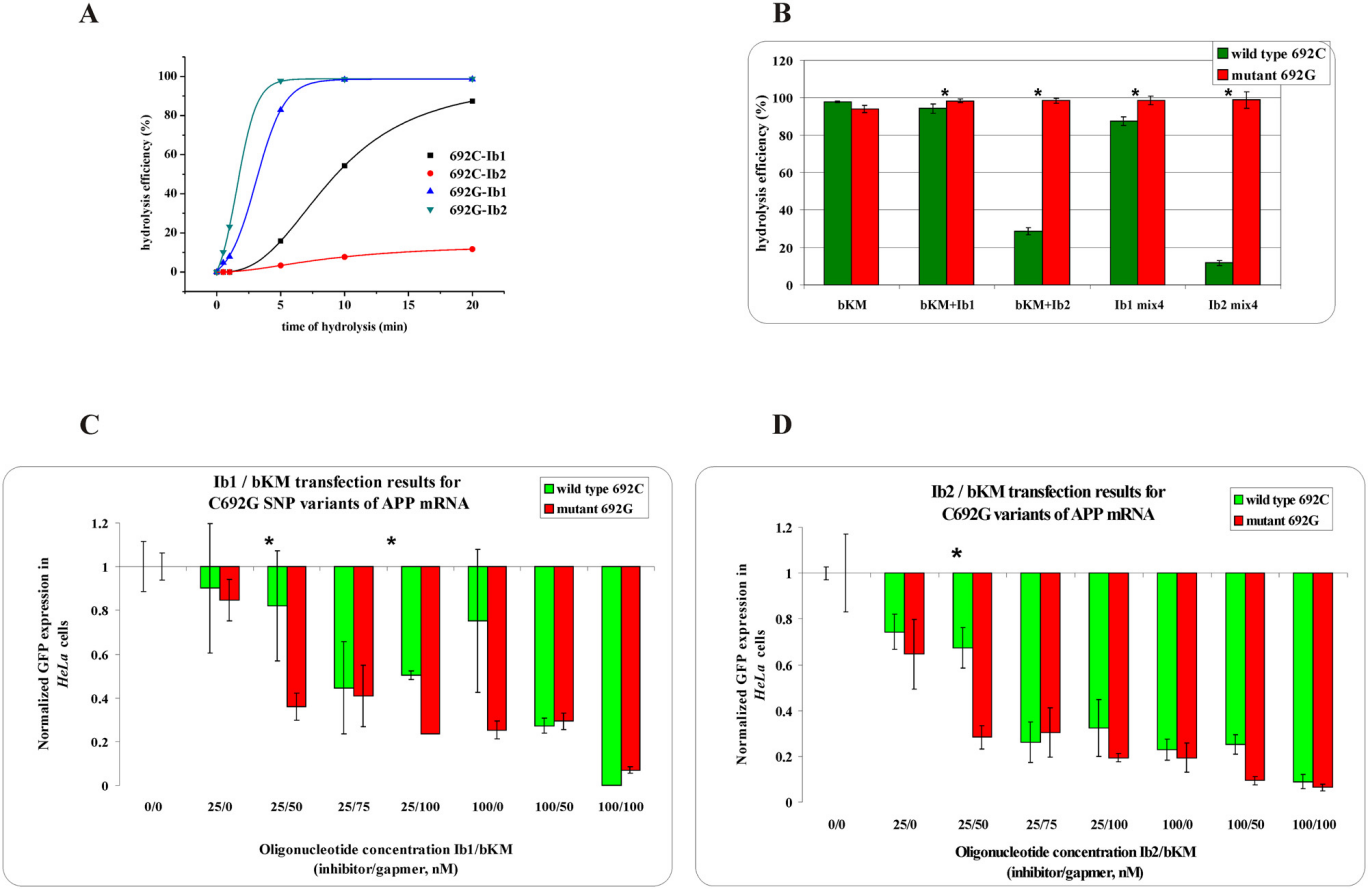
The RNase H assay results for C692G SNP within *APP* mRNA. ** (A)** kinetics of the WT and Mut RNAs cleavage in the presence of the gapmer (bKM) and the shorter (Ib1) or longer (Ib2) inhibitor, where black squares indicate degradation of WT RNA in the presence of the Ib1/bKM/Mut RNA mixture, red dots—degradation of WT RNA in the presence of the Ib2/bKM/ Mut RNA mixture, blue triangles—degradation of Mut RNA in the presence of the Ib1/bKM/WT RNA mixture and green triangles—degradation of Mut RNA in the presence of the Ib2/bKM/WT RNA mixture, **(B)** stability of the WT RNA (green bar) and Mut RNA (red bar) in the presence of: gapmer bKM only (first pair of bars from the left), gapmer bKM and short inhibitor Ib1 (second) or gapmer bKM and longer inhibitor Ib2 (third), and in the WT/Mut RNA/bKM/Ib1 mixture (fourth) and WT/Mut RNA/bKM/Ib2 mixture (fifth); statistically significant differences between the mean hydrolysis efficiency of the RNA variants (P<0.05) are marked with asterisk,**(C,D)** Results of *HeLa* cells cotransfection with WT/Mut C692G-pEGFP constructs and different amounts of inhibitor and gapmer antisense oligonucleotides. qPCR results of tandem approach with **(C)** shorter inhibitor Ib1, **(D)** Longer inhibitor Ib2. Statistically significant differences between the mean of the RNA variants expression (P<0.05) are marked with asterisk.

**Fig 4:**
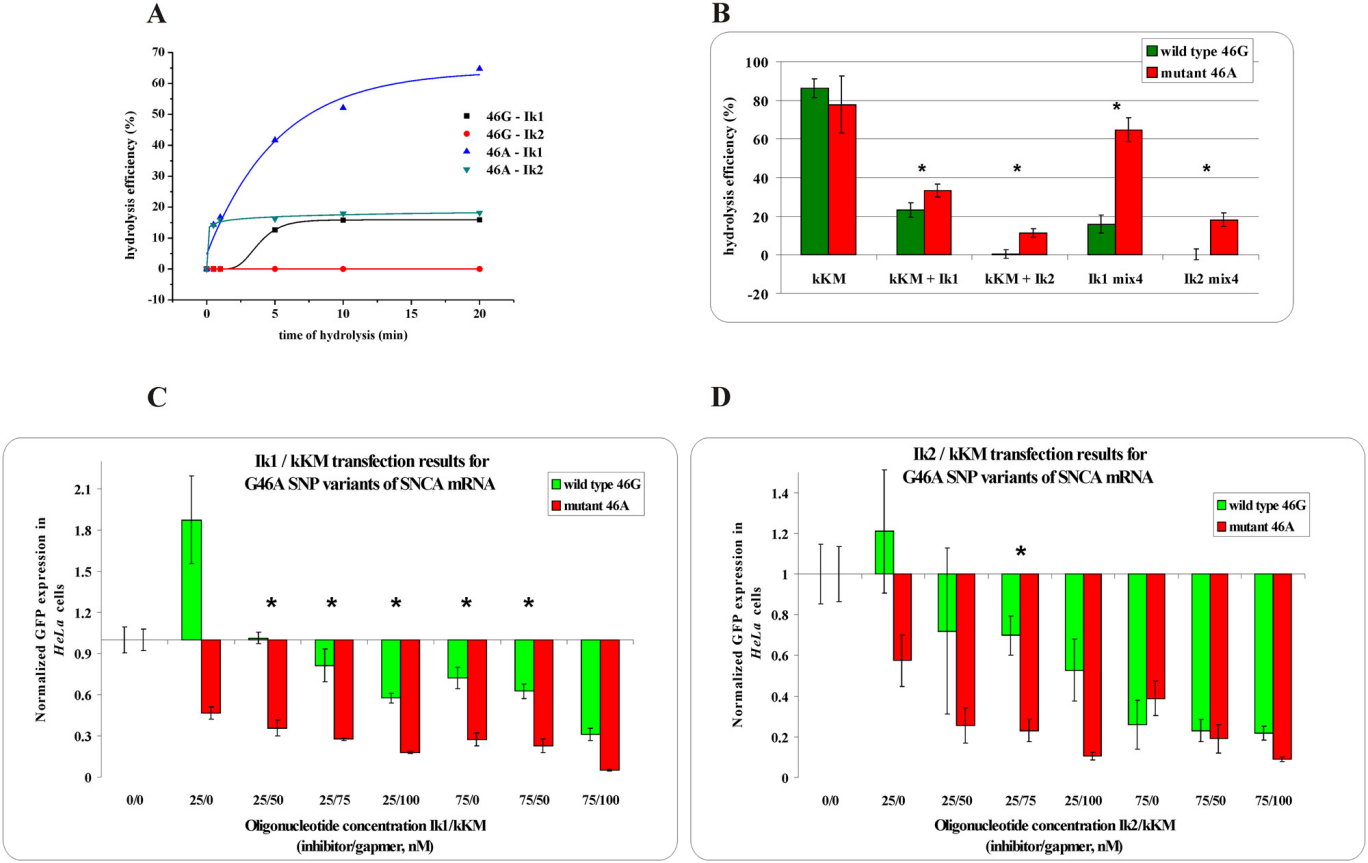
The RNase H assay results for G46A SNP within *SNCA* mRNA. ** (A)** kinetics of the WT and Mut RNAs cleavage in the presence of the gapmer (kKM) and the shorter (Ik1) or longer (Ik2) inhibitor, where black squares indicate degradation of WT RNA in the presence of the Ik1/kKM/Mut RNA mixture, red dots—degradation of WT RNA in the presence of the Ik2/kKM/ Mut RNA mixture, blue triangles—degradation of Mut RNA in the presence of the Ik1/kKM/WT RNA mixture and green triangles—degradation of Mut RNA in the presence of the Ik2/kKM/WT RNA mixture, **(B)** stability of the WT RNA (green bar) and Mut RNA (red bar) in the presence of: gapmer kKM only (first pair of bars from the left), gapmer kKM and short inhibitor Ik1 (second) or gapmer kKM and longer inhibitor Ik2 (third), and in the WT/Mut RNA/kKM/Ik1 mixture (fourth) and WT/Mut RNA/kKM/Ik2 mixture (fifth). Statistically significant differences between the mean hydrolysis efficiency of the RNA variants (P<0.05) are marked with asterisk,**(C,D)** Results of *HeLa* cells cotransfection with WT/Mut G46A -pEGFP constructs and different amounts of inhibitor and gapmer antisense oligonucleotides. qPCR results of tandem approach with **(C)** shorter inhibitor Ik1, **(D)** Longer inhibitor Ik2. Statistically significant differences between the mean of the RNA variants expression (P<0.05) are marked with asterisk.

**Fig 5:**
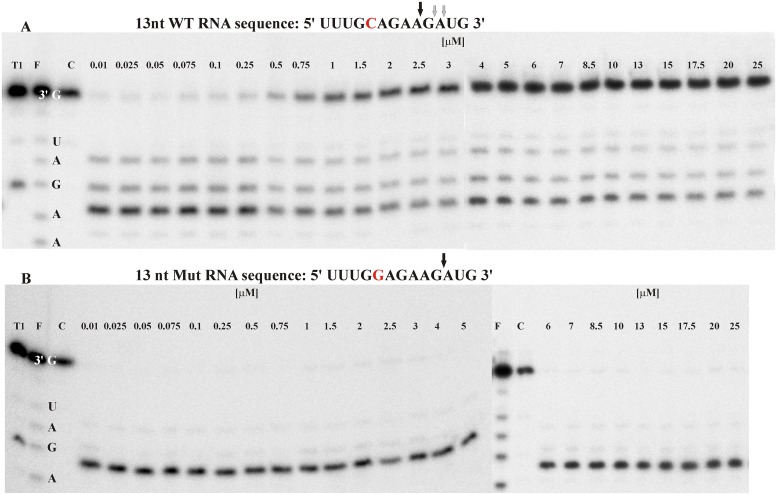
The influence of the shorter inhibitor Ib1 on the cleavage of RNA C692G transversion variants in the presence of bKM gapmer oligonucleotide. ** (A)** wild type RNA allele C **(B)** mutated RNA allele G. The sequence and the cleavage sites of analyzed RNAs are presented above gel images. The concentrations of WT and/or Mut RNA variants and the gapmer were 100 nM and 10 nM, respectively. The concentration of the Ib1 inhibitor ranged from 0.01 to 25 μM, as indicated by numbers above the lines. RNase H assay of designed model RNAs usually resulted in 1–2 main products of cleavage and a few minor products. The cleavage pattern is characteristic for particular duplex and could differ depending on presence or absence of a mismatch. Abbreviations in the gel picture mean as follow: T1- RNase T1 cleavage products, F—formamide hydrolysis products, C—control sample. The control sample contained all the mixture components exept for gapmer oligonucleotide.
