# Supplementary figures and images for: A Tandem Oligonucleotide Approach for SNP-Selective RNA Degradation Using Modified Antisense Oligonucleotides
Source: PLoS One. 2015 Nov 6;10(11):e0142139. doi: 10.1371/journal.pone.0142139 (PMC4704561; doi:10.1371/journal.pone.0142139)

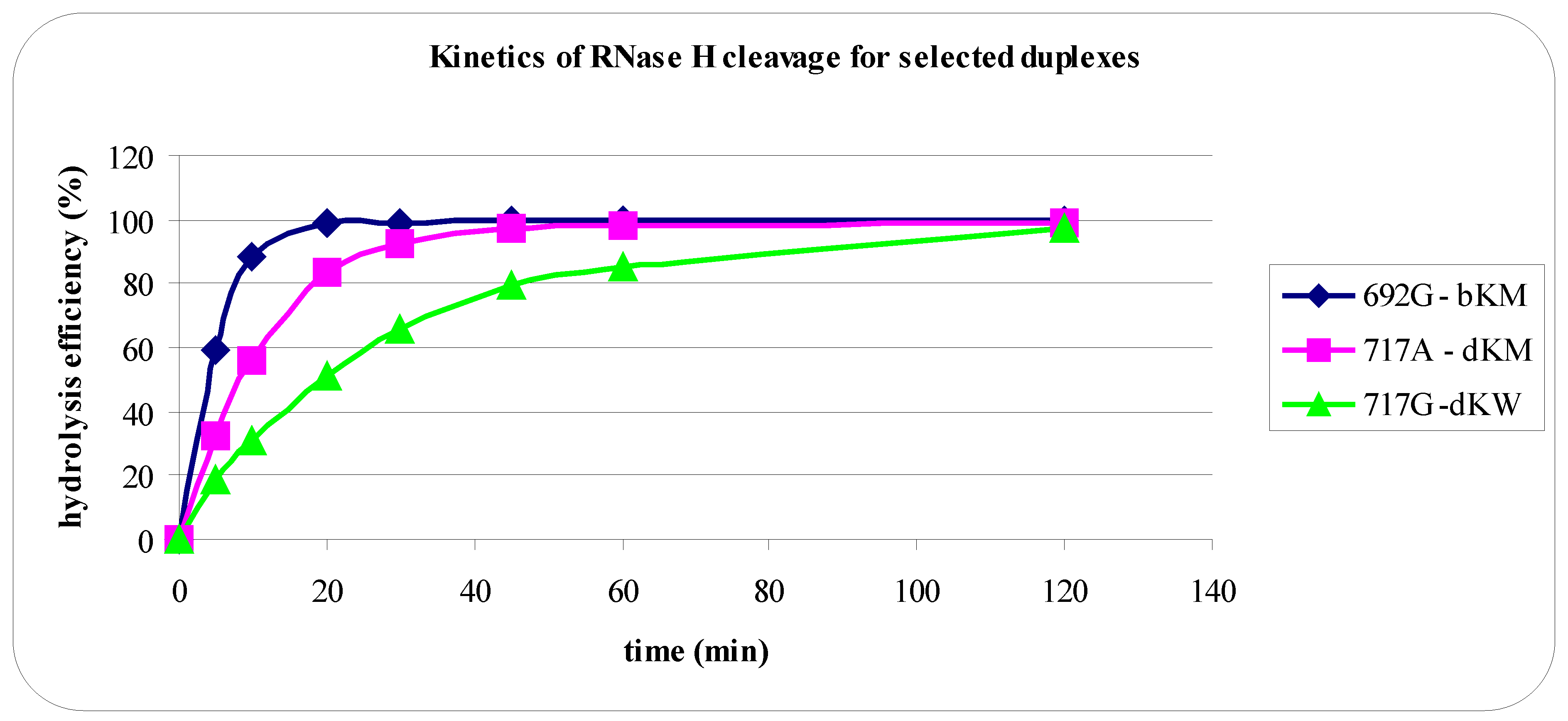

Supplement: S1 Fig — Most of the analyzed RNAs were totally degraded up to twenty minutes of RNase H hydrolysis. This could be seen also in the charts presenting RNase H hydrolysis efficiency depending on oligonucleotides content in the reaction mixture (Figs 3A, 4A, 5A and 6A and the upper charts in the S7 Fig. The exeption was additionally tested, as one of the controls, 717G-dKW duplex (WT RNA of APP G717A SNP with its complemetary gapmer antisense oligonucleotide dKW) which, probably because of the sequence, was digested five times slower. (TIF) [file pone.0142139.s001.tif]

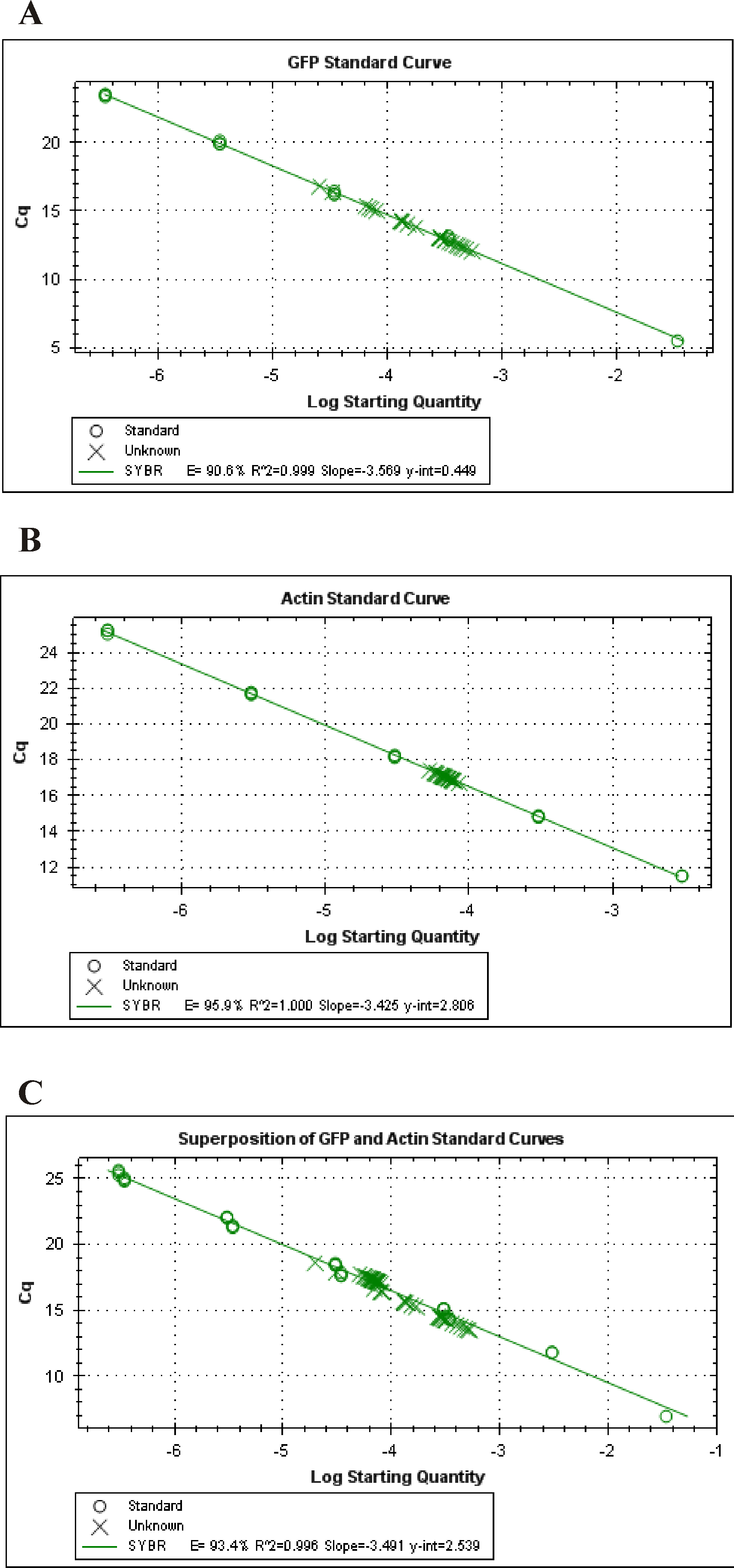

Supplement: S2 Fig — Standard curves for GFP (target, upper chart—A) β-actin (reference, middle chart—B), and their superposition for relative gene expression normalization (lower chart—C). Parameters of the curves were determined with Bio-Rad CFX Manager 3.0 software. As the standard curves for target and reference genes run in parallel, expression changes were determined with direct method based of comparison of the normalized target expression with the control sample, which usually was transfection of plasmid constructs only. (TIF) [file pone.0142139.s002.tif]

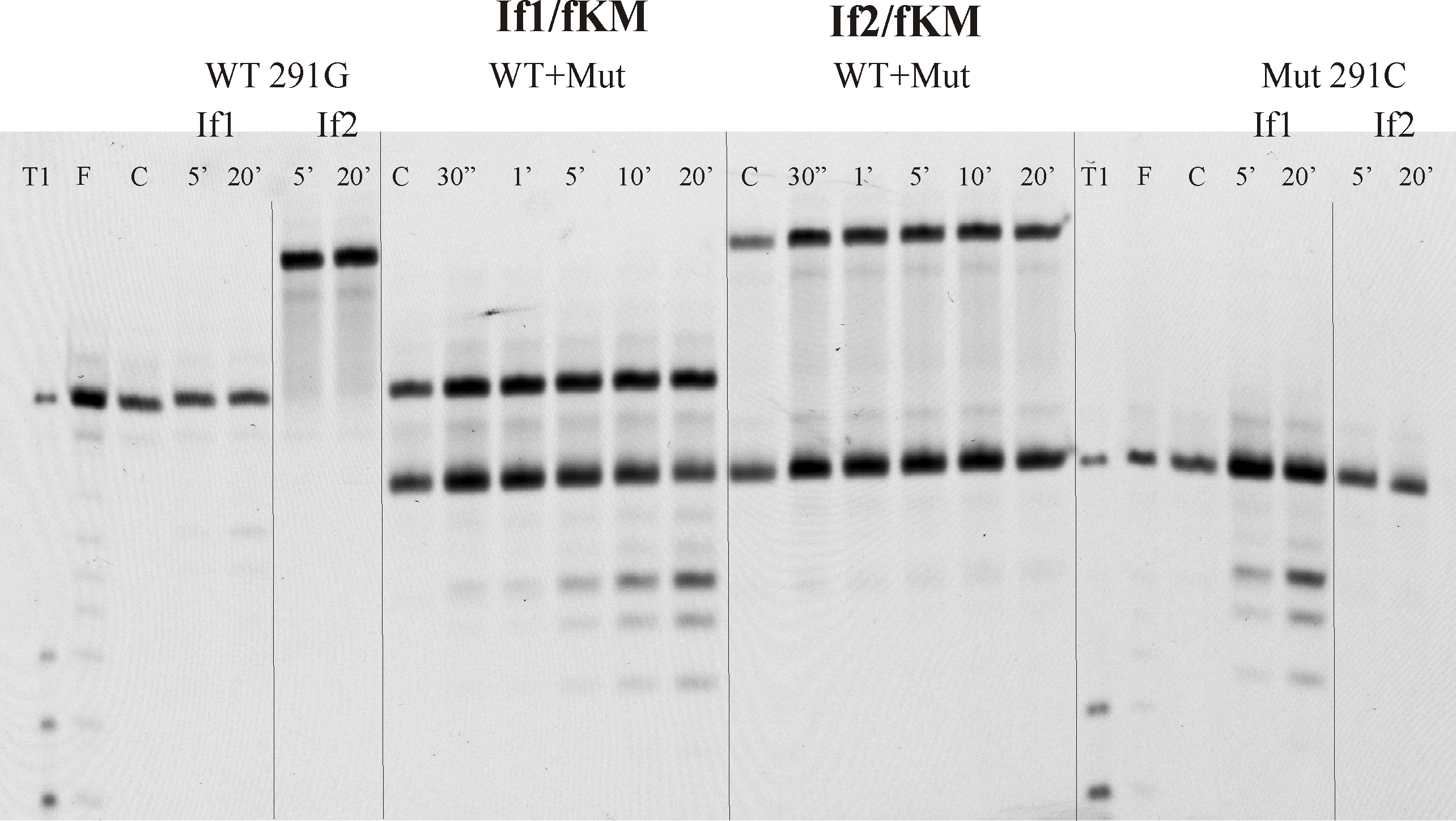

Supplement: S3 Fig — If2 inhibitor changes the WT RNA migration under denaturing gel conditions (stable duplex in 7M urea). (TIF) [file pone.0142139.s003.tif]

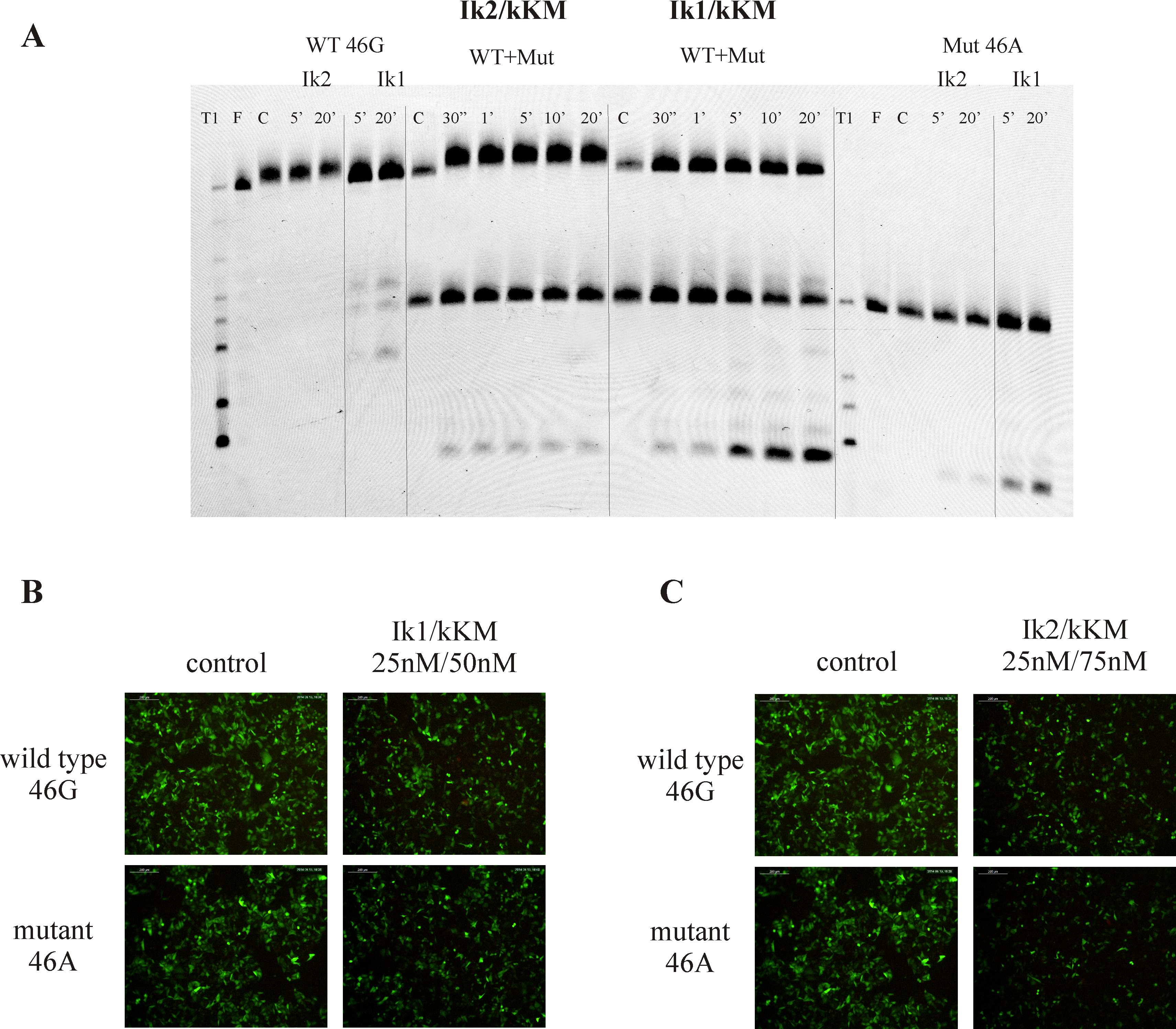

Supplement: S4 Fig — (A) Gel image after electrophoresis of G46A alleles cleavage of SNCA RNA with RNase H and Ik1/kKM or Ik2/kKM antisense oligonucleotides. (B) and (C) Fluorescence microscope images of HeLa cells (magnification 10x) after 24h cotransfection with WT/Mut G46A -pEGFP constructs and concentrations of inhibitor (shorter—B, and longer—C) and gapmer antisense oligonucleotides, that give statistically significant difference between both alleles expression. (TIF) [file pone.0142139.s004.tif]

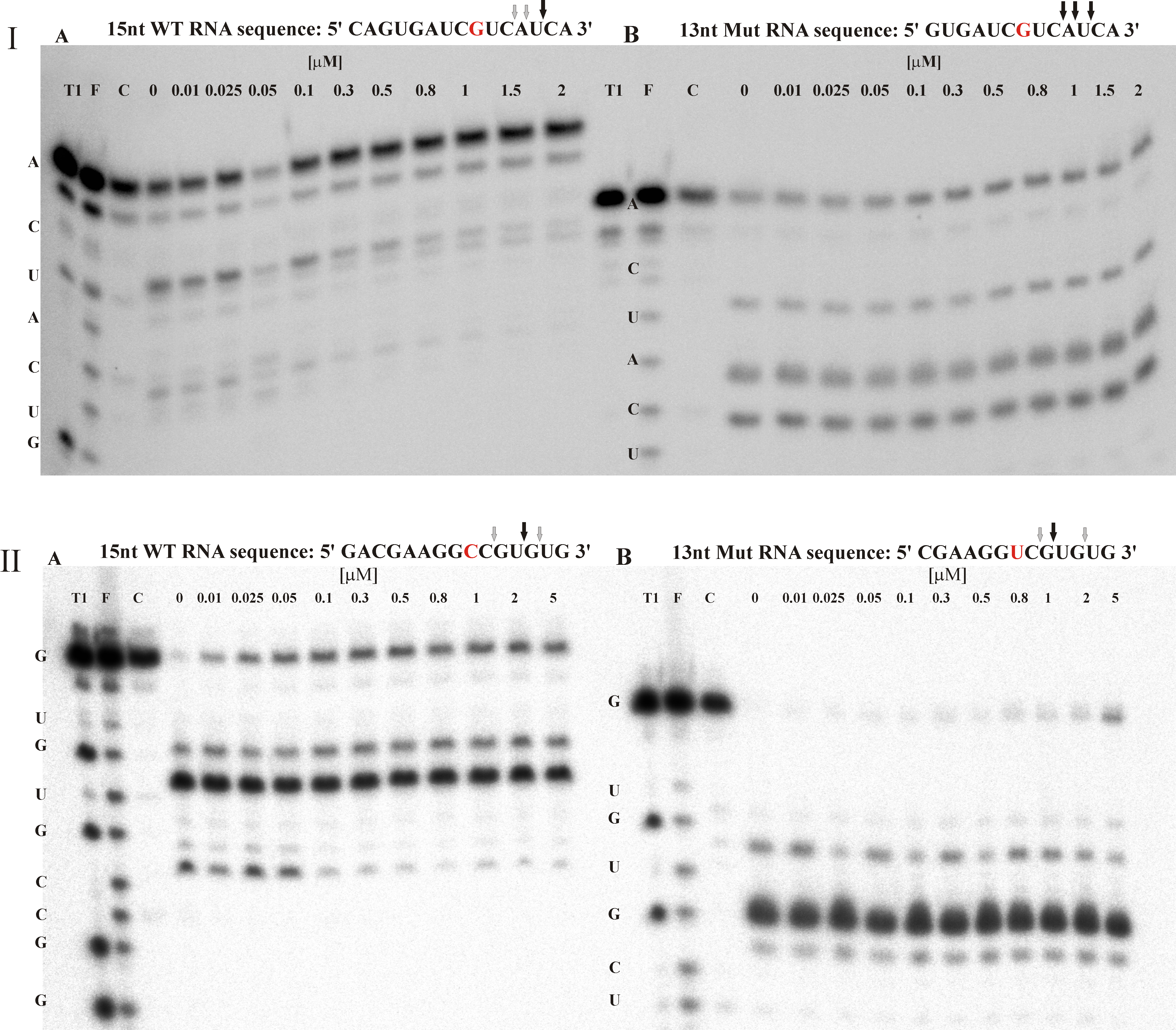

Supplement: S5 Fig — (A) model WT RNA, (B) model Mut RNA. Numbers above the path refer to inhibitor concentration (μM), arrows indicate cleavage sites. (TIF) [file pone.0142139.s005.tif]

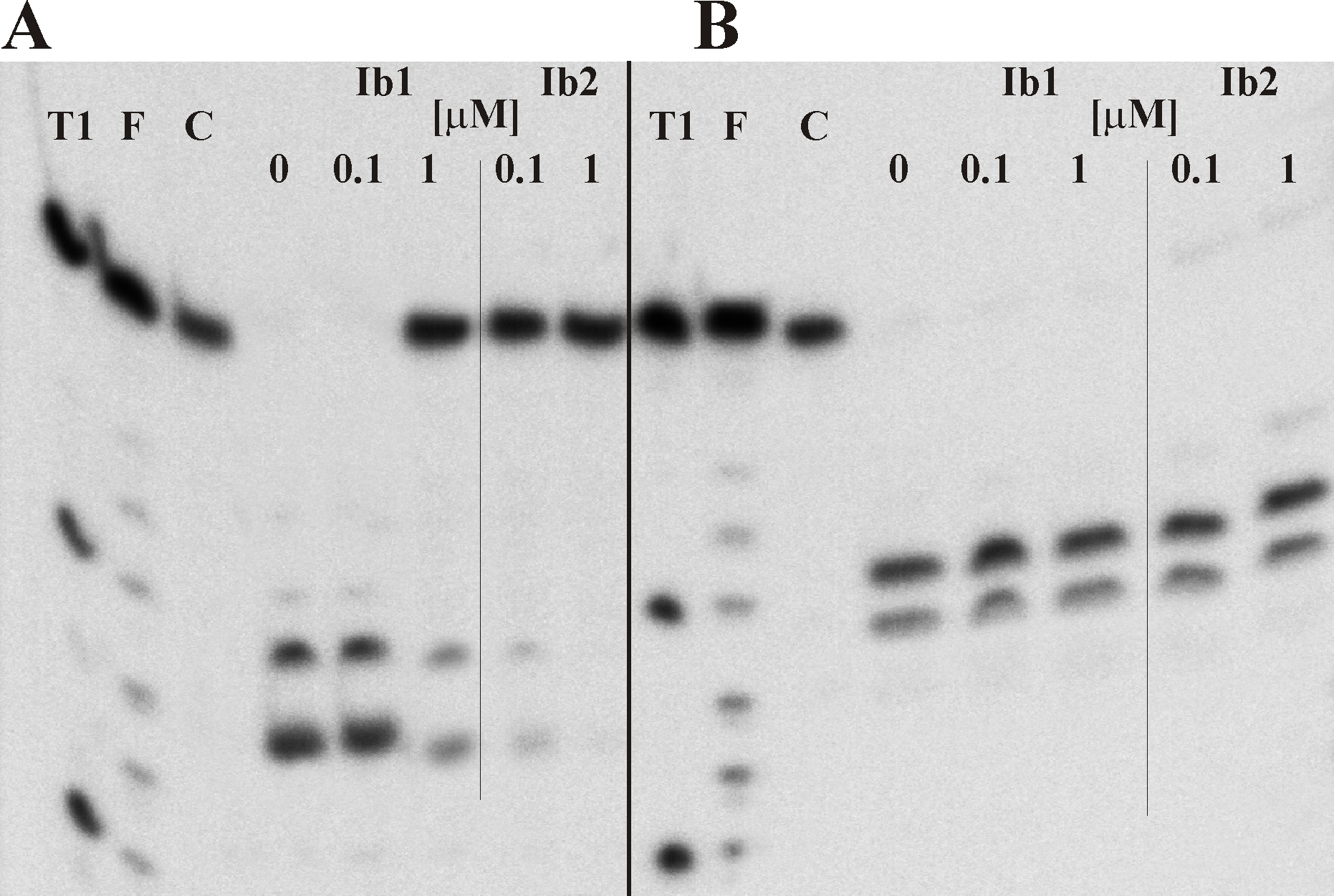

Supplement: S6 Fig — (A) WT 692C RNA and (B) Mutated 692G RNA. (TIF) [file pone.0142139.s006.tif]

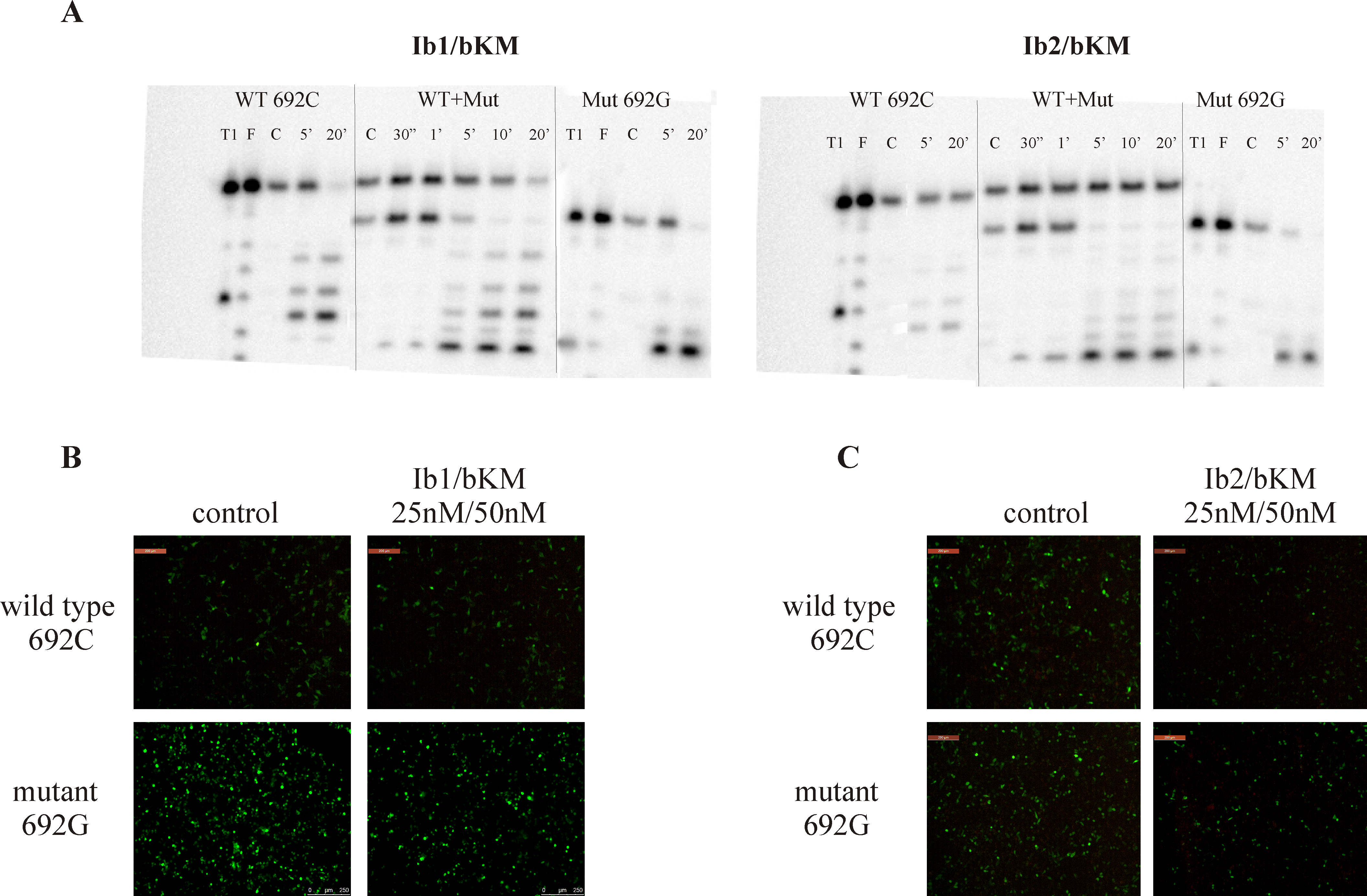

Supplement: S7 Fig — (B) and (C) Fluorescence microscope images of HeLa cells (magnification 10x) after 24h cotransfection with WT/Mut C692G -pEGFP constructs and concentrations of inhibitor (shorter—B, and longer—C) and gapmer antisense oligonucleotides, that give statistically significant difference between both alleles expression. (TIF) [file pone.0142139.s007.tif]

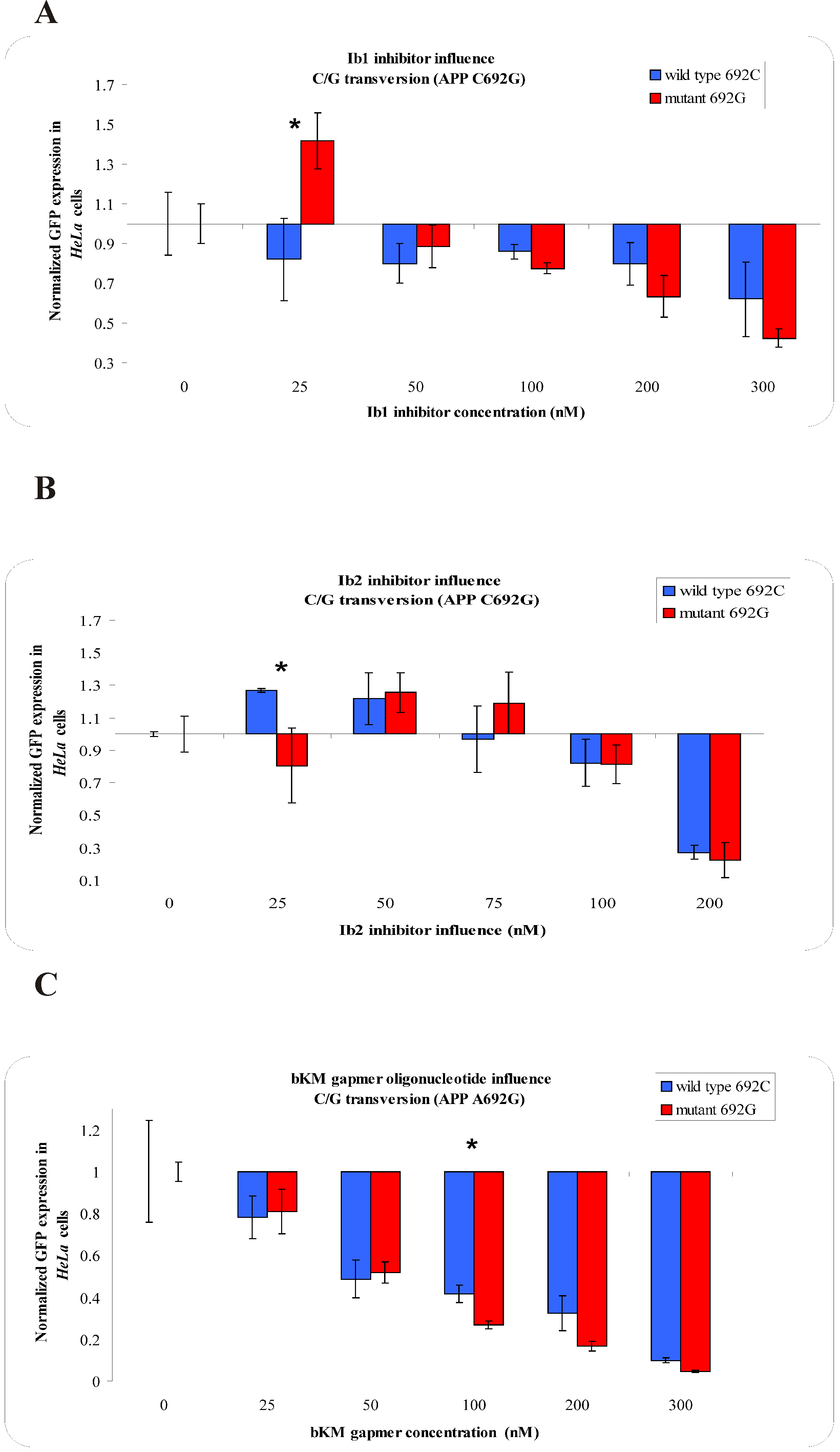

Supplement: S8 Fig — Statistically significant difference (P<0.05) are marked by asterisks. (TIF) [file pone.0142139.s008.tif]

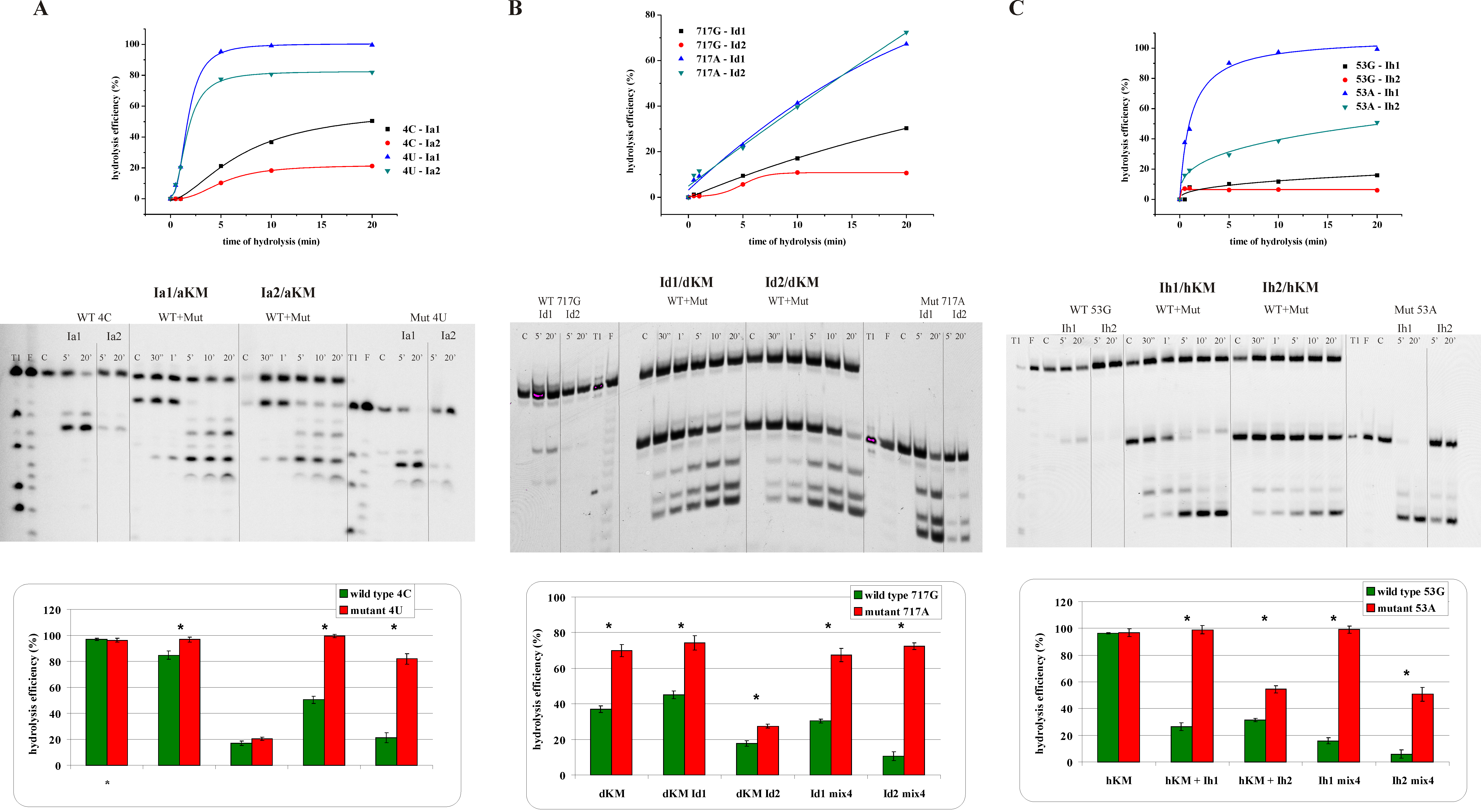

Supplement: S9 Fig — Upper charts present kinetics of RNase H hydrolysis, lower charts—efficiency of hydrolysis depending on presence of reaction mixture components. Statistically significant differences between mean hydrolysis efficiency of WT and Mut RNA cleavage are marked with asterisk (P<0.05, based on t-Student test) (TIF) [file pone.0142139.s009.tif]

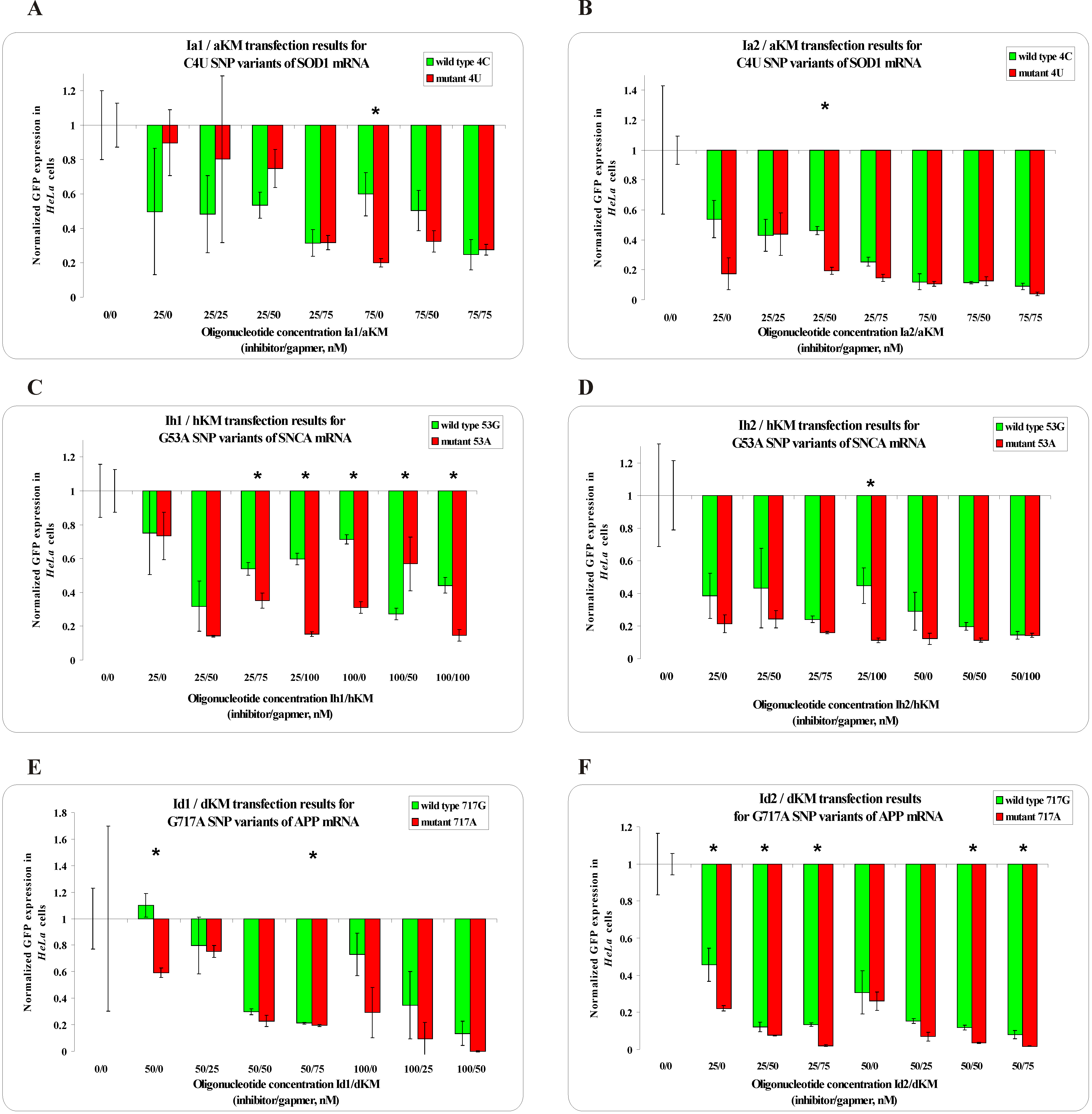

Supplement: S10 Fig — Statistically significant differences between mean normalized GFP expression of analyzed RNA variants are marked with asterisk (P<0.05) (TIF) [file pone.0142139.s010.tif]

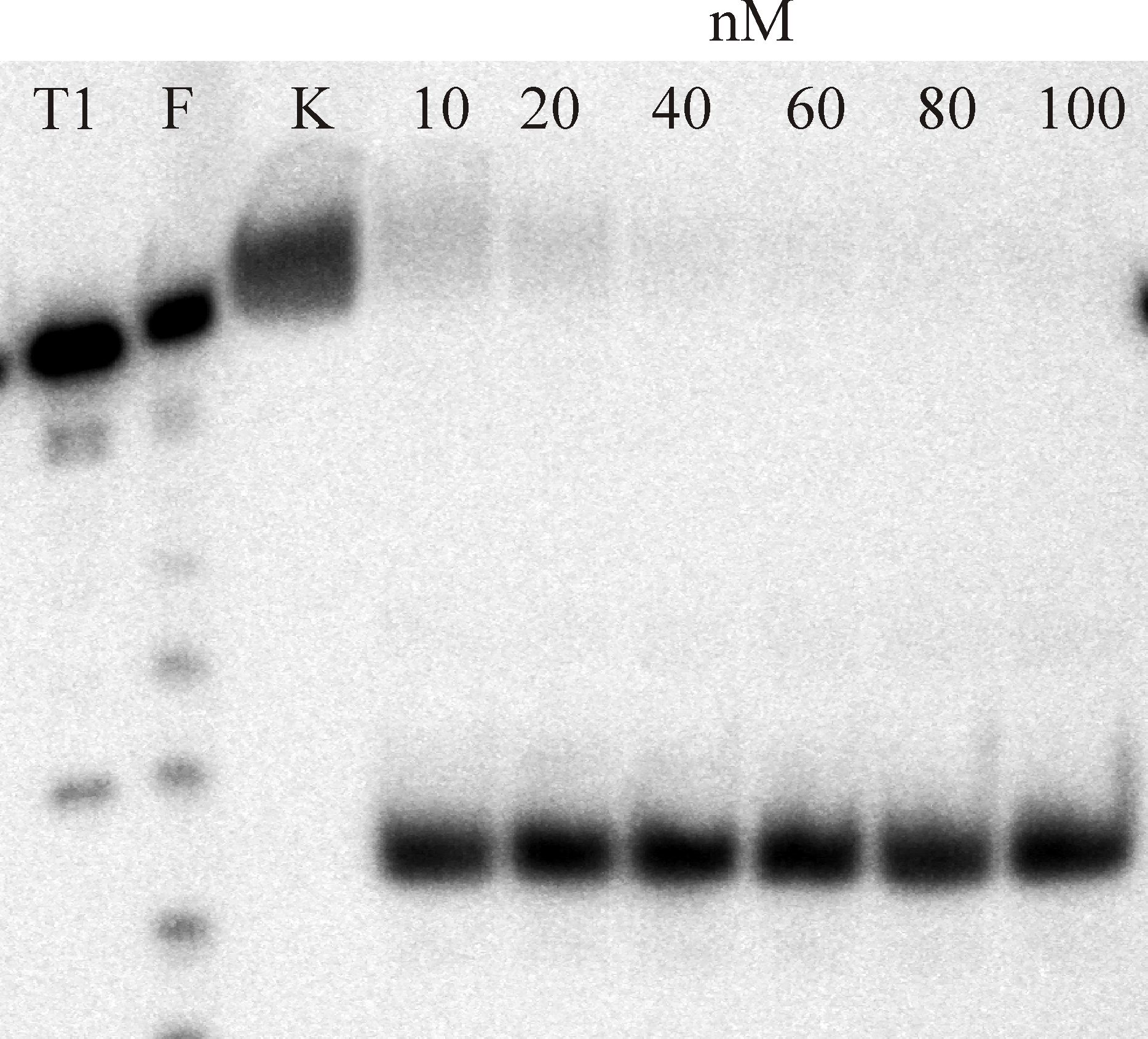

Supplement: S11 Fig — T1—RNase T1 cleavage of 692G RNA, F—formamide hydrolysis of 692G RNA, K—control sample without gapmer oligonucleotide (bKM concentration of 0) (TIF) [file pone.0142139.s011.tif]
