# Supplementary material for: A Tandem Oligonucleotide Approach for SNP-Selective RNA Degradation Using Modified Antisense Oligonucleotides
Source: PLoS One. 2015 Nov 6;10(11):e0142139. doi: 10.1371/journal.pone.0142139 (PMC4704561; doi:10.1371/journal.pone.0142139)
Supplement: S1 Table — (PDF) [file pone.0142139.s012.pdf]

Name	Length	5’-3’ sequence
Ia1	7	$A^{M}C^{M}G^{M}G^{L}C^{M}C^{M}U^{M}$
Ia2	10	$C^{M}A^{M}C^{M}G^{M}G^{L}C^{M}C^{M}U^{M}U^{M}C^{M}$
aKM	13	$C^{L}A^{M}C^{L}$**acgacct**$U^{L}C^{M}G^{L}$
Ib1	7	$U^{M}C^{M}U^{M}G^{L}C^{M}A^{M}A^{M}$
Ib2	10	$C^{M}U^{M}U^{M}C^{M}U^{M}G^{L}C^{M}A^{M}A^{M}A^{M}$
bKM	13	$C^{L}A^{M}U^{L}$**cttctcc**$A^{L}A^{M}A^{L}$
Id1	7	$U^{M}G^{M}A^{M}C^{L}G^{M}A^{M}U^{M}$
Id2	10	$G^{M}A^{M}U^{M}G^{M}A^{M}C^{L}G^{M}A^{M}U^{M}C^{M}$
dKM	13	$U^{L}G^{M}A^{L}$**tgatgat**$C^{L}A^{M}C^{L}$
Ie1	7	$U^{M}C^{M}U^{M}U^{L}C^{M}U^{M}G^{M}$
Ie2	9	$C^{M}A^{M}U^{M}C^{M}U^{M}U^{L}C^{M}U^{M}G^{M}$
eKM	13	$C^{L}A^{M}U^{L}$**ctcctgc**$A^{L}A^{M}A^{L}$
If1	7	$C^{M}C^{M}C^{M}C^{L}C^{M}U^{M}G^{M}$
If2	10	$G^{M}U^{M}C^{M}C^{M}C^{M}C^{L}C^{M}U^{M}G^{M}C^{M}$
fKM	13	$U^{L}A^{M}G^{L}$**gtcccgc**$U^{L}G^{M}C^{L}$
Ih1	7	$U^{M}U^{M}G^{M}C^{L}C^{M}A^{M}C^{M}$
Ih2	10	$U^{M}G^{M}U^{M}U^{M}G^{M}C^{L}C^{M}A^{M}C^{M}A^{M}$
hKM	13	$C^{L}U^{M}G^{L}$**ttgtcac**$A^{L}C^{M}C^{L}$
Ik1	7	$C^{M}C^{M}U^{M}C^{L}C^{M}U^{M}U^{M}$
Ik2	10	$C^{M}C^{M}C^{M}U^{M}C^{L}C^{M}U^{M}U^{M}G^{M}G^{M}$
kKM	13	$C^{L}U^{M}C^{L}$**ccttctt**$G^{L}G^{M}U^{L}$
