# Supplementary material for: A Tandem Oligonucleotide Approach for SNP-Selective RNA Degradation Using Modified Antisense Oligonucleotides
Source: PLoS One. 2015 Nov 6;10(11):e0142139. doi: 10.1371/journal.pone.0142139 (PMC4704561; doi:10.1371/journal.pone.0142139)
Supplement: S2 Table — (PDF) [file pone.0142139.s013.pdf]

WT RNA/ASO duplexes (5'-3')/(3'-5')	$-\Delta G^{\circ}_{37}$	Tm[a]	Mut RNA/ASO duplexes (5'-3')/(3'-5')	$-\Delta G^{\circ}_{37}$	Tm[a]	Inhibitor[b,c] influence $\Delta\Delta G^{\circ}_{37}$	WT-Mut RNA $\Delta\Delta G^{\circ}_{37}$	Percentage hydrolysis of RNA *in vitro*[d]			Expression of RNA in *HeLa* cells		
Transversion C/G (C692G, APP gene)								WT RNA	Mut RNA	Difference	WT RNA	Mut RNA	Selectivity
UUUGCAGAAGAUG<br>AAACGUCU Ib1	9.29±0.10	53.1	UUUGGAGAAGAUG<br>AAACGUCU	4.60±-0.18	22.8	0[b]	4.69	87.4 ± 2.4[e]<br>(10.5 ± 4.5)[f]	98.6 ± 1.8[e]<br>(-4.60 ± 6.0)[f]	11.2 ± 3.0	82%	36%	46% (25/50)[j]
UUUGCAGAAGAUG<br>AAACGUCUUC Ib2	12.60±0.07	66.9	UUUGGAGAAGAUG<br>AAACGUCUUC	8.64±0.03	46.4	(0)[c]	3.96	11.7 ± 2.5[g]<br>(86.2 ± 4.5)[h]	98.8 ± 4.9[g]<br>(-4.80 ± 7.5)[h]	87.1 ± 5.50	67%	28%	39%<br>(25/50)
UUUGCAGAAGAUG<br>AAACCTCTTCUAC bKM	9.81±0.08	48.6	UUUGGAGAAGAUG<br>AAACCTCTTCUAC	17.92±0.59	67.2	8.63[b]<br>(5.32)[c]	-8.11	97.9 ± 3.8[i]<br>(0.0)	94.0 ± 5.7[i]<br>(0.0)	-3.9 ± 6.85	49%	52%	3%<br>(50)[k]
Transition G/A (G717A, APP gene)													
GUGAUCGUCAUCA<br>UAGCAGU Id1	9.31±0.03	56.1	GUGAUCAUCAUCA<br>UAGCAGU	7.71±0.04	44.4	0	1.60	30.3 ± 7.7<br>(27.4 ± 17.6)	67.3 ± 2.9<br>(16.3 ± 13.0)	37.0 ± 8.2	-	-	-
GUGAUCGUCAUCA<br>CUAGCAGUAG Id2	15.01±0.15	72.9	GUGAUCAUCAUCA<br>CUAGCAGUAG	10.36±0.10	53.5	(0)	4.65	10.7 ± 3.2<br>(47.7 ± 16.8/	72.4 ± 3.8<br>(11.2 ± 13.1)	61.7 ± 5.0	-	-	-
GUGAUCGUCAUCA<br>CACTAGTAGTAGU dKM	10.48±0.05	57.3	GUGAUCAUCAUCA<br>CACTAGTAGTAGU	10.98±0.09	61.3	1.67<br>(-4.03)	-0.50	57.7 ± 15.8<br>(0.0)	83.6 ± 12.6<br>(0.0)	25.9 ± 20.8	123%	31%	92%<br>(50)
Transition A/G (A693G, APP)													
UUUGCAGAAGAUG<br>GUCUUCU Ie1	9.24±0.04	51.6	UUUGCAGGAGAUG<br>GUCUUCU	7.52±0.01	43.0	0	1.72	78.2 ± 1.9<br>(18.7 ± 2.5)	99.6 ± 0.7<br>(-1.9 ± 2.4)	21.4 ± 2.0	-	-	-
UUUGCAGAAGAUG<br>GUCUUCUAC Ie2	11.88±0.09	58.7	UUUGCAGGAGAUG<br>GUCUUCUAC	10.44±0.06	52.0	(0)	1.44	14.3 ± 2.1<br>(82.6 ± 2.7)	56.6 ± 2.6<br>(41.1± 3.5)	42.3 ± 3.3	50%	5%	45%<br>(25/100)
UUUGCAGAAGAUG<br>AAACGTCCTCUAC eKM	11.69±0.15	57.3	UUUGCAGGAGAUG<br>AAACGTCCTCUAC	17.00±0.48	73.2	8.76<br>(5.12)	-5.31	96.9 ± 1.7<br>(0.0)	97.7 ± 2.3<br>(0.0)	0.8 ± 3.3	80%	37%	43%<br>(50)
Transition G/A (G46A, SNCA)													
ACCAAGGAGGGAG<br>UUCCUCC Ik1	11.83±0.10	67.9	ACCAAGAAGGGAG<br>UUCCUCC	7.76±0.02	63.4	0	4.07	15.9 ± 1.2<br>(70.4 ± 4.9)	64.8 ± 3.4<br>(13.0 ± 15.0)	48.9 ± 3.9	100%	35%	65%<br>(25/50)
ACCAAGGAGGGAG<br>GGUUCCUCCC Ik2	20.98±0.58	82.8	ACCAAGAAGGGAG<br>GGUUCCUCCC	13.45±0.17	63.5	(0)	7.53	0	18.2 ± 4.4<br>(59.6 ± 15.4)	18.2	70%	23%	47%<br>(25/75)
ACCAAGGAGGGAG<br>UGGTTCTTCCCUC kKM	20.59±0.50	78.8	ACCAAGAAGGGAG<br>UGGTTCTTCCCUC	23.95±0.43	81.3	12.12<br>(2.97)	-3.36	86.3 ± 4.8<br>(0.0)	77.8 ± 14.6<br>(0.0)	-8.5 ± 15.4	28%	17%	10%<br>(50)
Transition G/A (G53A, SNCA)													
GGUGUGGCAACAG<br>CACCGUU Ih1	10.33±0.07	60.4	GGUGUGACAACAG<br>CACCGUU	6.22±0.05	34.2	0	4.11	15.9 ± 2.0<br>(81.0 ± 2.9)	99.3 ± 0.8<br>(-0.2 ± 0.9)	83.4 ± 2.1	60%	15%	45%<br>(25/100)
GGUGUGGCAACAG<br>ACACCGUUGU Ih2	16.12±0.13	75.1	GGUGUGACAACAG<br>ACACCGUUGU	10.58±0.06	54.1	(0)	5.54	5.9 ± 2.3<br>(91.0 ± 3.1)	50.8 ± 3.4<br>(48.3 ± 3.5)	44.9 ± 4.1	45%	11%	34%<br>(25/100)
GGUGUGGCAACAG<br>CCACACTGTTGUC hKM	16.29±0.09	72.9	GGUGUGACAACAG<br>CCACACTGTTGUC	18.99±0.34	73.8	8.61<br>(2.87)	-2.90	96.9 ± 2.1<br>(0.0)	99.1 ± 0.3<br>(0.0)	2.2 ± 2.3	46%	30%	16%<br>(100)
Transition C/U (C4U, SOD1)													
CGAAGGCCGUGUG<br>UCCGGCA Ia1	12.41±0.12	71.8	CGAAGGUCGUGUG<br>UCCGGCA	10.12±0.19	56.1	0	2.29	50.5 ± 3.1<br>(46.0 ± 4.5)	99.6 ± 1.3<br>(-5.7 ± 7.9)	49.1 ± 3.4	50%	32%	18%<br>(75/50)
CGAAGGCCGUGUG<br>CUUCCGGCAC Ia2	17.04±0.65	80.1	CGAAGGUCGUGUG<br>CUUCCGGCAC	16.56±0.09	69.5	(0)	0.48	21.3 ± 1.6<br>(75.0 ± 3.7)	81.9 ± 2.0<br>(12.0 ± 8.0)	60.6 ± 2.6	46%	19%	27%<br>(25/50)
CGAAGGCCGUGUG<br>GCUTTCAGCACAC aKM	17.62±0.34	71.2	CGAAGGUCGUGUG<br>GCUTTCAGCACAC	20.24±0.15	78.7	7.83<br>(3.20)	-2.62	96.3 ± 3.3<br>(0.0)	93.9 ± 7.4<br>(0.0)	-2.4 ± 8.1	110%	55%	55%<br>(50)
Transversion G/C (G291C, SCA3)													
GCAGGGGGACCUA<br>GUCCCCC If1	14.41 ± 1.13	68.8	GCAGCGGGACCUA<br>GUCCCCC	6.47 ± 0.05	36.5	0	7.94	11.7 ± 2.3<br>(58.4 ± 3.4)	31.1 ± 1.9<br>(2.6 ± 2.1)	19.4 ± 3.0	-	-	-
GCAGGGGGACCUA<br>CGUCCCCCUG If2	20.91 ± 0.87	82.7	GCAGCGGGACCUA<br>CGUCCCCCUG	11.35 ± 0.07	59.2	(0)	9.56	0	2.7 ± 1.5<br>(31.0 ± 1.9)	2.7	-	-	-
GCAGGGGGACCUA<br>CGUCGCCCTGGAU fKM	17.94 ± 0.75	66.5	GCAGCGGGACCUA<br>CGUCGCCCTGGAU	27.7 ± 2.9	80.3	13.29<br>(6.79)	-9.76	70.1 ± 2.5<br>(0.0)	33.7 ± 1.1<br>(0.0)	-36.4 ± 2.7	79%	13%	66%<br>(50)

a – melting temperature of the duplex at concentration 10^{-4}M, b – differences in thermodynamic stability of the WT RNA/short inhibitor (Ix1) and Mut RNA/gapmer (xKM) duplexes, c– differences in thermodynamic stability of WT RNA/longer inhibitor (Ix2 and Mut RNA/gapmer (xKM) duplexes, d – errors calculated with standard deviation, e – for cleavage of WT or Mut RNA shorter inhibitors and gapmer were used, f - differences in hydrolysis of WT or Mut RNA in presence of gapmer only and the appropriate RNA in the presence of both the gapmer and shorter inhibitor, g – for cleavage of WT or Mut RNA the longer inhibitor and gapmer were used, h – differences in hydrolysis of WT or Mut RNA in the presence of gapmer only and the appropriate RNA in the presence of both the gapmer and longer inhibitor, i – for cleavage of WT or Mut RNA only the gapmer was used, j – the abbreviation 25/50 refers to transfection of the HeLa cells with 25 nM and 50 nM concentrations of inhibitor and gapmer, respectively, k – the abbreviation 50 refers to transfection of the *HeLa* cells with 50 nM of gapmer only, empty cells (-) – statistically insignificant results were obtained. Underlined letters indicated the position of the SNP, red letters corresponded to 2'-O-methylated RNA, blue letters indicated the position of the LNA nucleotides, the bolded region corresponded to the DNA fragment within the gapmer, and the remaining letters correspond to the RNA.
