# Supplementary material for: A Tandem Oligonucleotide Approach for SNP-Selective RNA Degradation Using Modified Antisense Oligonucleotides
Source: PLoS One. 2015 Nov 6;10(11):e0142139. doi: 10.1371/journal.pone.0142139 (PMC4704561; doi:10.1371/journal.pone.0142139)
Supplement: S3 Table — (PDF) [file pone.0142139.s014.pdf]

| | | **Kd (nM)** | | |
|---|---|---|---|---|
| Gene | SNP type wt/mutant (codon) | Wild type | Mutant | EMSA result |
| APP | C/G (692) | 180.2 ± 6.4 | 60.9 ± 5.1 | 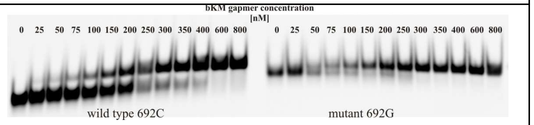 |
| | A/G (693) | 233.1 ± 2.5 | 442.0 ± 49.2 | 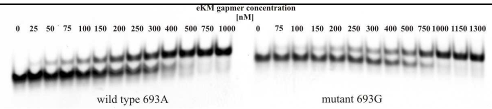 |
| | G/A (717) | 342.1 ± 49.2 | 419.3 ± 49.5 | 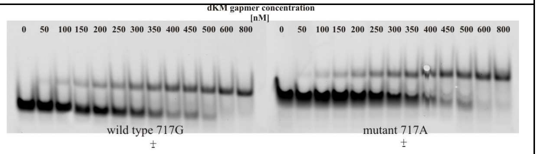 |
| SNCA | G/A (46) | 42.9 ± 1.4 | 60.9 ± 3.6 | 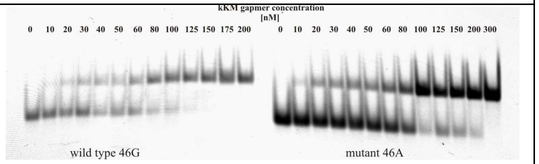 |
| | G/A (53) | 130.8 ± 2.0 | 197.3 ± 50.0 | 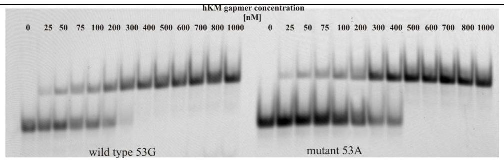 |
| SOD1 | C/U (4) | 89.0 ± 1.3 | n/a | 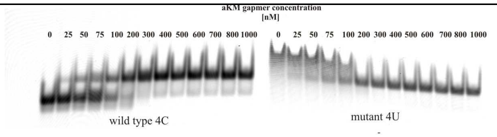 |
| SCA3 | G/C (291) | n/a | n/a | - |
