# Supplementary material for: A Tandem Oligonucleotide Approach for SNP-Selective RNA Degradation Using Modified Antisense Oligonucleotides
Source: PLoS One. 2015 Nov 6;10(11):e0142139. doi: 10.1371/journal.pone.0142139 (PMC4704561; doi:10.1371/journal.pone.0142139)
Supplement: S4 Table — (PDF) [file pone.0142139.s015.pdf]

Variant	Name	Length	5’-3’ sequence
WT	291G	13	GCAG**G**GGGACCUA
Mut	291C	13	GCAG**C**GGGACCUA
WT	692C	13	UUUG**C**AGAAGAUG
Mut	692G	13	UUUG**G**AGAAGAUG
WT	693A	13	UUUGCAG**A**AGAUG
Mut	693G	13	UUUGCAG**G**AGAUG
WT	717G	13	GUGAUC**G**UCAUCA
Mut	717A	13	GUGAUC**A**UCAUCA
WT	4C	13	CGAAGG**C**CGUGUG
Mut	4U	13	CGAAGG**U**CGUGUG
WT	53G	13	GGUGUG**G**CAACAG
Mut	53A	13	GGUGUG**A**CAACAG
WT	46G	13	ACCAAG**G**AGGGAG
Mut	46A	13	ACCAAG**A**AGGGAG
